# Cross-sectional associations between 24-hour activity behaviours and motor competence in youth: a compositional data analysis

**DOI:** 10.1186/s44167-022-00003-3

**Published:** 2022-09-01

**Authors:** Richard Tyler, Andrew J. Atkin, Jack R. Dainty, Dorothea Dumuid, Stuart J. Fairclough

**Affiliations:** 1 Health Research Institute and Movement Behaviours, Nutrition, Health, and Wellbeing Research Group, Department of Sport and Physical Activity, Edge Hill University, Ormskirk, UK; 2 School of Health Sciences, Faculty of Medicine and Health Sciences, University of East Anglia, Norwich, UK; 3 Alliance for Research in Exercise, Nutrition and Activity, Allied Health & Human Performance, University of South Australia, Adelaide, SA Australia; 4 Murdoch Children’s Research Institute, Parkville, VIC Australia

**Keywords:** Physical activity, Sedentary behaviour, Sleep, Motor skills, Children, Adolescents, Time-use epidemiology

## Abstract

**Background:**

The study aimed to examine the cross-sectional associations between 24-h activity compositions and motor competence in children and adolescents, while stratifying by sex and school type (primary or secondary school) and estimate differences in motor competence associated with reallocations of time between activity behaviours.

**Methods:**

Data were collected from 359 participants (aged 11.5 ± 1.4 years; 49.3% boys; 96.9% White British). Seven-day 24-h activity behaviours [sleep, sedentary time, light physical activity (LPA), moderate-to-vigorous physical activity (MVPA)] were assessed using wrist-worn accelerometers. Motor competence outcomes were obtained using the Dragon Challenge (process, product, time, and overall scores). Linear mixed models examined associations between activity behaviour compositions and motor competence outcomes for all participants and stratified by school type (primary or secondary) and sex. Post-hoc analyses modelled the associations of reallocating fixed durations of time between activity behaviours with the outcomes.

**Results:**

In all participants, relative to other activity behaviours, MVPA had the strongest associations with motor competence outcomes. Time reallocations (5, 10, 15, 20 min) to MVPA from any of the other three behaviours were associated with higher overall, process, and time scores [effect sizes (ES) = 0.05–0.07 (5 min) and 0.19–0.27 (20 min)]. The stratified models displayed that MVPA had the strongest associations with outcomes in both sexes, irrespective of school type. The largest positive, and negative estimated differences occurred when MVPA hypothetically replaced LPA or sleep [ES = 0.04–0.10 (5 min) and 0.14–0.39 (20 min)], and when LPA or sleep hypothetically replaced MVPA [ES = − 0.03 to − 0.11 (5 min) and − 0.13 to − 0.54 (20 min)], respectively.

**Conclusions:**

Relative to other activity behaviours, MVPA had the strongest association overall with motor competence outcomes. Hypothetical reallocations of time from LPA or sleep to MVPA (and vice versa) were associated with the largest positive estimated differences in motor competence outcomes. Therefore, our findings reinforce the key role of MVPA for children’s and adolescents’ motor competence.

**Supplementary Information:**

The online version contains supplementary material available at 10.1186/s44167-022-00003-3.

## Practical implications

- This study provides empirical evidence that more MVPA, relative to other activity behaviours, is associated with positive motor competence outcomes.
- Our findings reinforce the key role of MVPA for children and adolescents’ motor competence and highlight the collective association of activity behaviours with motor competence outcomes, which could guide the focus of motor competence interventions.
- Promoting and protecting opportunities for MVPA is beneficial for motor competence in children and adolescents and practical approaches to increase MVPA are advocated.

## Background

Childhood and adolescence are critical periods and provide a window of opportunity for the development of motor competence [1]. Motor competence is an umbrella term used to represent an individual’s ability to perform skilfully on a wide range of motor tasks [1–5]. The term encapsulates fundamental, combined, and complex movement skills, which are amalgamated to form general, refined, and specific movement patterns, and utilised to perform goal-directed movements/motor tasks in various physical activity (PA) and sporting pursuits [1–6]. Therefore, it is purported that motor competence is an important precursor for increasing positive health trajectories, particularly PA, across the lifespan [4, 5, 7, 8].

Previous studies and systematic reviews have reported evidence for positive associations between motor competence and PA levels [2, 7], and that a positive feedback loop or reciprocal relationship exists, in which youth with greater levels of PA, develop better motor competence, leading to further increases in PA engagement [2, 5, 7, 8]. However, a recent systematic review revealed that most previous studies did not find a significant prospective association between PA and subsequent motor competence [3]. The authors suggest that, among other reasons, the common placement of device-measured (i.e., accelerometer) PA at the waist, may not capture the intensity of discrete skilled movements, such as throwing, meaning that the association between PA and motor competence will likely be low [3]. Therefore, there is a need to investigate associations between device-measured PA and motor competence using accelerometry worn at alternative locations, such as the wrist. An additional reason for the lack of association between PA and motor competence may be that previous research has focused on time allocated to moderate-to-vigorous physical activity (MVPA) and its association to motor competence in isolation, or only with partial adjustment for time in other physical behaviours (i.e., sedentary time (ST), light physical activity (LPA), and sleep) [2, 3, 7]. Accounting for the inter-relations of MVPA, LPA, ST, and sleep is important because these activity behaviours are constrained to the 24-h of the day. Thus, a change in one behaviour results in change in others (e.g., increase in ST could reflect a decrease in MVPA) [9], and therefore the association between motor competence and a specific behaviour also depends on other behaviours.

More recently, studies using compositional data analyses have investigated associations between activity behaviour compositions and motor competence; specifically, fundamental movement skill proficiency, in pre-school and primary school-aged children [10–13], without stratifying by sex. The stratification by sex may be important given the sex differences in preferences for engaging in different types of PA throughout childhood and adolescence, and that these different activity types could have diverging roles in developing motor competence [7]. In addition, none of these compositional studies have investigated motor competence using proficiency in combinations of fundamental, combined, and complex movement skills in children and adolescents. Given that the preferences for engaging in different types and intensities of PA may change throughout childhood (primary school) and adolescence (secondary school), and these differences may have differing contributions towards the development of movement skills and motor competence [2, 7], stratification by school type (primary and secondary school) warrants investigation.

Previous studies on this topic have used primarily process-based assessments of motor competence [10–13]. Given that individual process- and product-based assessments shine a light on different and limited aspects of motor competence [1], hybrid-based motor competence assessments (using both a process- and product-based approach) have been developed for children and adolescents [1, 6]. Thus, investigating associations between activity behaviour compositions and motor competence using a measure that utilises a hybrid-based approach to provide a more holistic view of motor competence, is warranted.

Reflecting on this, we aimed to (i) examine the cross-sectional associations between 24-h activity compositions and motor competence (using a hybrid-based measure of motor competence) in children and adolescents while stratifying by sex and school type, and (ii) estimate differences in motor competence associated with reallocations of time between activity behaviours. The current study fits within Research Area Two: outcomes of health-related time-use compositions (specifically, associations between time-use compositions and physical skills/abilities) of the framework for Viable Integrative Research in Time-Use Epidemiology (VIRTUE) [14].

## Methods

Following institutional ethical approval (#SPA-REC-2018-007) written informed parent consent and participant assent were obtained from 382 students from ten primary and two secondary schools in the West Lancashire region of northwest England. From these, 359 students (23 were absent on data collection days) took part in the study between April 2019 and March 2020, as part of a wider project described elsewhere [15].

Participants’ ethnicity, dates of birth, and home postal codes were obtained from the schools’ information management systems and used to calculate decimal age and 2019 Indices of Multiple Deprivation (IMD) deciles [16]. Height and body mass were measured using a portable stadiometer (Seca 213, Seca Ltd, Birmingham, UK) and calibrated scales (Seca 813, Seca Ltd, Birmingham, UK), respectively. From these, body mass index (BMI) and BMI z-scores were calculated using BMI reference curves for the UK [17], and age- and sex-specific IOTF BMI cut-points were used to classify weight status [18].

To obtain activity behaviours, participants wore triaxial accelerometers (ActiGraph GT9X, ActiGraph, Pensacola, FL, USA), set at 100 Hz, on the non-dominant wrist for 24 h·day^−1^ over 7 days. Data were processed in R (http://cran.r-project.org) using GGIR (v1.9.0) [19], which performed auto-calibration procedures, identified non-wear, and converted raw triaxial accelerometer signals into 1 omnidirectional measure of acceleration (Euclidean Norm Minus-One; ENMO) [15, 20, 21]. ENMO values were averaged per 5 s epoch over the 7 monitored days to represent average acceleration expressed as milligravitational units (mg). Participants were excluded if accelerometer post-calibration error was > 10 mg and if < 3 valid days of wear (i.e., ≥ 16 h·day^−1^) were recorded [15]. Youth-specific non-dominant wrist ENMO cut-points of 50 mg and 200 mg [22, 23], defined estimated ST/LPA, and MVPA, respectively. Sleep duration was estimated using a polysomnography-validated accelerometer algorithm [24].

Participants undertook the Dragon Challenge (DC) assessment of motor competence, involving nine tasks (Balance Bench, Core Agility, Wobble Spot, Overarm Throw, Basketball Dribble, Catch, Jumping Patterns, T-Agility, and Sprint) completed in a timed circuit [6]. Each DC task requires participants to apply a different combination of fundamental, combined, and complex movement skills [6]. Good validity and reliability of the assessment have been established [6]. Using standardised methodology [6], scoring was completed in situ, by an expert assessor with > 100 h of DC training/in situ experience [with good intra- and inter-rater reliability against other expert assessors, using pre-recorded DC reliability videos (all ICCs > 0.9)]. The assessor had no prior knowledge of participants’ movement capabilities. Participants were assessed against criteria on the process (quality of movement technique) and product (successfully achieving the outcome/goal) for each task, and time taken to complete the circuit (hybrid-based approach) [6]. From these, process, product, time, and overall scores were calculated (motor competence outcomes), with larger scores indicative of higher levels of motor competence [6].

Imputation was performed for missing data (24.5% of activity behaviour data; 0.3% BMI z-score; 2.5% IMD decile; 1.1% DC scores) in IBM SPSS Statistics (v25, IBM Inc., NY, USA), using the expectation maximisation algorithm [15]. Compositional analyses were conducted using R v3.6.2 (www.r-project.org) [15]. Sets of pivot coordinates (time-use composition expressed as four specific sets of three isometric log-ratio (ILR) coordinates) were used as explanatory variables in regression analyses [15]. A mixed model approach (‘school’ as the single, random intercept) analysed the association of DC scores with the time-use ILRs [15], whilst adjusting for age, sex, BMI z-scores, and IMD deciles [10–13]. Additional sex- and school-stratified analyses were performed.

Models were checked to ensure assumptions for the use of linear mixed effects were met. The ANOVA table of the model fit displayed whether the set of time-use ILRs was significantly associated with the selected DC score. If significant, four models were carried out, each using a different set of pivot co-ordinates, so that they examined associations with one activity behaviour, relative to all remaining behaviours. To determine the most dominant behaviour in the relationship with the DC scores, the regression coefficient and *p*-value of the first coordinate (ILR_1) representing one behaviour relative to remaining behaviours was examined [15]. The most dominant behaviour was then the focus of compositional isotemporal substitution analyses to model the associations of reallocating fixed time durations (5, 10, 15, 20 min) between it and the other behaviours [9]. Effect-sizes (ES) for estimated differences in DC scores were expressed in terms of the model residual standard deviation [15, 25].

## Results

Mean and standard deviations of the measured variables and geometric means of activity behaviours (linearly adjusted to add up to 1440 min per day) are presented in Table 1. Participants were predominantly White British (96.9%), of medium-to-high socioeconomic position (IMD decile 7.3 ± 2.3), 49.3% were boys, and 24.6% were overweight or obese. There was high compliance to wearing the accelerometers (5.9 ± 1.6 days of valid wear for 22.8 ± 1.0 h·day^−1^). Participants spent 44.1% of the 24-h day in ST, 37% in sleep, 14.6% in LPA, and 3.5% in MVPA (calculated from geometric mean of each behaviour). Only 23.1% of participants averaged ≥ 60 min MVPA per day [26] and 72.4% had < 9 h sleep per night [27]. Primary school participants spent more time in sleep, LPA, and MVPA, and less in ST than their older peers*.* Dragon Challenge scores for participants aligned to published national/normative data [6]; the participants did not have high levels of motor competence nor particularly low levels. The secondary school participants performed better in DC scores than their younger peers.

**Table 1:** Descriptive characteristics of study participants and descriptive accelerometer and motor competence variables (mean (SD) unless indicated otherwise)

Variables	All schools	Primary schools (all)	Secondary schools (all)	All schools (girls)	Primary schools (girls)	Secondary schools (girls)	All schools (boys)	Primary schools (boys)	Secondary schools (boys)
n	359	210	149	182	103	79	177	107	70
Age (years)	11.5 (1.4)	10.4 (0.7)	13.0 (0.3)	11.5 (1.5)	10.3 (0.7)	13.1 (0.3)	11.5 (1.3)	10.5 (0.7)	13.0 (0.3)
Girls (%)	50.7	49.0	53.0	–	–	–	–	–	–
Ethnicity									
White British (%)	96.9	97.1	96.6	98.9	99.0	98.7	94.9	95.3	94.3
Socioeconomic status									
IMD score	13.0 (8.9)	11.3 (6.5)	15.4 (11.0)	13.3 (9.9)	11.6 (7.1)	15.4 (12.4)	12.7 (7.7)	11.0 (5.8)	15.3 (9.3)
IMD decile	7.3 (2.3)	7.8 (2.0)	6.7 (2.5)	7.4 (2.4)	7.7 (2.1)	6.8 (2.6)	7.3 (2.2)	7.8 (1.9)	6.5 (2.3)
Height (cm)	149.1 (11.3)	143.0 (8.3)	157.8 (9.1)	149.0 (11.7)	142.4 (8.6)	157.6 (9.6)	149.3 (10.9)	143.6 (8.0)	158.1 (8.6)
Body mass (kg)	43.1 (12.2)	37.9 (9.7)	50.4 (11.6)	43.6 (12.9)	38.0 (10.4)	51.0 (12.2)	42.5 (11.5)	37.8 (9.2)	49.7 (10.9)
BMI (kg m^−2^)	19.1 (3.6)	18.3 (3.2)	20.1 (3.8)	19.4 (3.9)	18.5 (3.4)	20.5 (4.1)	18.8 (3.3)	18.1 (3.0)	19.7 (3.4)
BMI z-score	0.39 (1.21)	0.38 (1.23)	0.41 (1.17)	0.35 (1.23)	0.35 (1.24)	0.35 (1.23)	0.44 (1.18)	0.42 (1.22)	0.47 (1.12)
Weight status									
Underweight (%)	7.9	8.2	7.5	8.3	8.7	7.7	7.3	7.5	7.1
‘Normal’ weight (%)	67.6	66.7	68.9	66.9	64.1	70.5	68.4	69.2	67.1
Overweight (%)	19.8	20.5	18.9	18.2	20.4	15.4	21.5	20.6	22.9
Obese (%)	4.7	4.8	4.7	6.6	6.8	6.4	2.8	2.8	2.9
Accelerometer^a^									
ST (min·day^−1^)	635.8	580.6	693.6	639.6	592.8	701.0	632.7	598.9	684.8
LPA (min·day^−1^)	210.1	211.2	205.6	210.8	212.8	206.8	208.4	210.5	204.1
MVPA (min·day^−1^)	50.9	60.1	41.6	44.9	52.6	36.3	57.0	63.3	48.4
Meet PA guideline^b^ (%)	23.1	30.0	13.4	15.9	21.4	8.9	30.5	38.3	18.6
Sleep (min·day^−1^)	543.2	588.1	499.3	544.6	581.7	495.9	541.9	567.3	502.8
Meet sleep guideline^c^ (%)	27.6	41.4	8.1	32.4	51.5	7.6	22.6	31.8	8.6
Valid wear time (hour·day^−1^)	22.8 (1.0)	22.4 (0.8)	23.4 (0.9)	22.9 (1.0)	22.4 (0.8)	23.5 (0.8)	22.7 (1.0)	22.3 (0.8)	23.3 (0.9)
Number of days with valid wear time	5.9 (1.6)	6.1 (1.0)	5.6 (2.1)	6.1 (1.4)	6.2 (1.0)	6.0 (1.7)	5.6 (1.8)	6.0 (1.1)	5.1 (2.4)
Dragon Challenge scores									
Overall score	32.0 (7.4)	31.2 (7.4)	33.2 (7.4)	30.8 (7.2)	30.1 (6.7)	31.8 (7.7)	33.3 (7.5)	32.3 (7.9)	34.8 (6.7)
Process score	9.4 (3.2)	9.0 (3.1)	9.9 (3.2)	8.8 (3.2)	8.4 (2.9)	9.3 (3.5)	9.9 (3.1)	9.5 (3.2)	10.5 (2.9)
Product score	10.2 (3.5)	10.0 (3.5)	10.4 (3.6)	9.8 (3.6)	9.7 (3.4)	10.0 (3.8)	10.6 (3.5)	10.3 (3.6)	10.9 (3.3)
Time score	12.5 (2.1)	12.2 (2.2)	12.9 (1.8)	12.2 (1.8)	12.0 (1.8)	12.5 (1.8)	12.8 (2.2)	12.5 (2.5)	13.3 (1.8)

*IMD* Indices of Multiple Deprivation; *BMI* body mass index; *ST* sedentary time; *LPA* light physical activity; *MVPA* moderate-to-vigorous physical activity; *PA* physical activity

^a^Each behavioural time-use set was transformed to compositional means (expressed as the geometric mean of each behaviour, linearly adjusted to collectively sum to 1440 min)

^b^Minimum of 60 min·day^−1^ MVPA averaged over the week [26]

^c^Minimum of 9 h·night^−1^ sleep [27]

Dragon Challenge scores ranges were: overall score 0–54, process score 0–18, product score 0–18, time score 0–18

Table 2 summarises the results from the regression models and displays the significant associations between the activity composition ILR coordinates and DC scores, stratified by sex and school type (see Additional file 1: S1 for full model outputs).

**Table 2:** Associations between activity composition and motor competence outcomes

Motor competence outcomes	All schools		Primary schools (all)		Secondary schools (all)		All schools (girls)		Primary schools (girls)		Secondary schools (girls)		All schools (boys)		Primary schools (boys)		Secondary schools (boys)	
Dragon Challenge scores	*Χ*^2^	*p*	*Χ*^2^	*p*	*Χ*^2^	*p*	*Χ*^2^	*p*	*Χ*^2^	*p*	*Χ*^2^	*p*	*Χ*^2^	*P*	*Χ*^2^	*p*	*Χ*^2^	*p*
Overall score	**17.20**	** < 0.001**	4.21	0.24	**17.59**	** < 0.001**	**13.66**	** < 0.001**	4.76	0.19	**11.86**	**0.008**	**12.94**	** < 0.001**	**11.69**	**0.008**	7.33	0.06
Process score	**23.80**	** < 0.001**	3.74	0.29	**23.00**	** < 0.001**	**15.46**	** < 0.001**	3.30	0.35	**15.38**	**0.002**	**14.57**	** < 0.001**	**8.72**	**0.03**	**8.66**	**0.03**
Product score	6.19	0.10	0.90	0.83	**9.38**	**0.02**	**9.51**	**0.02**	**9.56**	**0.02**	**8.93**	**0.03**	6.25	0.10	**8.16**	**0.04**	2.97	0.40
Time score	**8.87**	**0.03**	**10.84**	**0.01**	5.31	0.15	2.04	0.56	4.26	0.23	1.06	0.79	**8.01**	**0.045**	**10.52**	**0.01**	5.82	0.12

Results from the regression models for each motor competence outcome: associations between the activity composition and Dragon Challenge scores, stratified by sex and school type. Activity composition expressed as isometric log-ratio coordinates. Models adjusted for age (excluding stratified by school type analysis), sex (excluding stratified by sex analyses), BMI z-score, and IMD decile. Bolded estimates are significant at *p* < 0.05 (‘school’ as the single, random intercept to account for nesting)

*Χ*^*2*^ chi-squared values that are generated by the statistical model (mixed effects)

Beta coefficients for the first pivot coordinate of the models showing significant associations between activity composition and outcomes are presented in Table 3. For all participants, MVPA relative to the other activity behaviours, was associated with positive estimated differences in overall, process and time scores. Among primary school participants, MVPA relative to the other behaviours was associated with better time score. Among secondary school participants, MVPA relative to the other behaviours was associated with positive estimated differences in overall and process scores, whilst, sleep, relative to the other behaviours, was associated with a lower product score. The sex-stratified models showed that for all girls (all schools combined) and for secondary school girls, sleep relative to the other activity behaviours was associated with poorer overall and product scores, and MVPA relative to the other behaviours was associated with positive estimated differences in process scores. For primary school girls, LPA relative to the other behaviours was associated with a better product score. For all boys, MVPA relative to other activity behaviours was associated with positive estimated differences in overall, process, and time scores. Furthermore, MVPA relative to other behaviours was associated with better time score and process score, in primary school boys and secondary school boys, respectively. Finally, in primary school boys, LPA relative to other behaviours was associated with worse overall, process, and product scores.

**Table 3:** Relationship between motor competence outcomes that were significantly associated with the activity composition, and the activity behaviour isometric log-ratio regression coefficients

Motor competence outcomes	*β*_1_ (SE)	*β*_1_ (SE)	*β*_1_ (SE)	*β*_1_ (SE)
Dragon Challenge scores	*ilr*1 (Sleep)	*ilr*1 (ST)	*ilr*1 (LPA)	*ilr*1 (MVPA)
All schools				
Overall score	− 2.45 (2.25)	− 0.60 (1.80)	− 1.76 (2.64)	**4.81 (1.37)**
Process score	− 1.00 (0.96)	− 0.52 (0.77)	− 0.86 (1.13)	**2.40 (0.59)**
Time score	− 0.43 (0.64)	− 0.03 (0.51)	− 0.58 (0.75)	**1.04 (0.39)**
Primary schools (all)				
Time score	1.52 (1.22)	− 1.54 (1.05)	− 1.72 (1.04)	**1.74 (0.64)**
Secondary schools (all)				
Overall score	− 7.34 (2.92)	− 0.26 (2.22)	3.06 (4.21)	**4.54 (1.87)**
Process score	− 2.63 (1.29)	− 0.50 (0.98)	0.45 (1.86)	**2.68 (0.83)**
Product score	**− 3.17 (1.52)**	0.11 (1.15)	1.48 (2.19)	1.58 (0.97)
All schools (girls)				
Overall score	**− 8.33 (4.29)**	− 0.05 (3.92)	5.78 (3.69)	2.60 (1.89)
Process score	− 3.48 (1.87)	0.09 (1.70)	1.67 (1.62)	**1.73 (0.83)**
Product score	**− 4.72 (2.23)**	0.75 (2.04)	3.39 (1.92)	0.57 (0.98)
Primary schools (girls)				
Product score	− 6.07 (3.43)	1.70 (2.82)	**7.71 (2.53)**	− 3.34 (1.60)
Secondary schools (girls)				
Overall score	**− 14.33 (6.16)**	3.76 (6.43)	5.86 (7.03)	4.72 (2.82)
Process score	− 5.64 (2.75)	0.94 (2.87)	1.79 (3.14)	**2.90 (1.26)**
Product score	**− 7.69 (3.18)**	3.11 (3.31)	2.71 (3.62)	1.86 (1.45)
All schools (boys)				
Overall score	0.56 (2.88)	− 0.26 (2.08)	− 7.28 (3.72)	**6.98 (2.02)**
Process score	0.20 (1.19)	− 0.34 (0.86)	− 2.86 (1.54)	**3.00 (0.83)**
Time score	− 0.55 (0.88)	0.08 (0.64)	− 1.19 (1.14)	**1.67 (0.62)**
Primary schools (boys)				
Overall score	9.97 (5.52)	− 4.65 (4.86)	**− 12.67 (5.01)**	7.34 (3.11)
Process score	2.82 (2.29)	− 0.94 (2.00)	**− 4.81 (2.08)**	2.93 (1.29)
Product score	5.59 (2.63)	− 2.34 (2.31)	**− 5.13 (2.38)**	1.88 (1.48)
Time score	1.99 (1.75)	− 1.63 (1.50)	− 2.95 (1.58)	**2.59 (0.99)**
Secondary schools (boys)				
Process score	− 1.81 (1.50)	− 0.63 (0.97)	0.23 (2.37)	**2.20 (1.15)**

*ST* sedentary time; *LPA* light physical activity; *MVPA* moderate-to-vigorous physical activity

*β*_1_ (*SE*), *ilr*1 (….): first isometric log-ratio regression coefficients from regression models represents one activity behaviour relative to all remaining behaviours (standard error in parentheses). Bolded coefficients indicate those significantly associated with the outcomes at *p* < 0.05

**Fig. 1:**
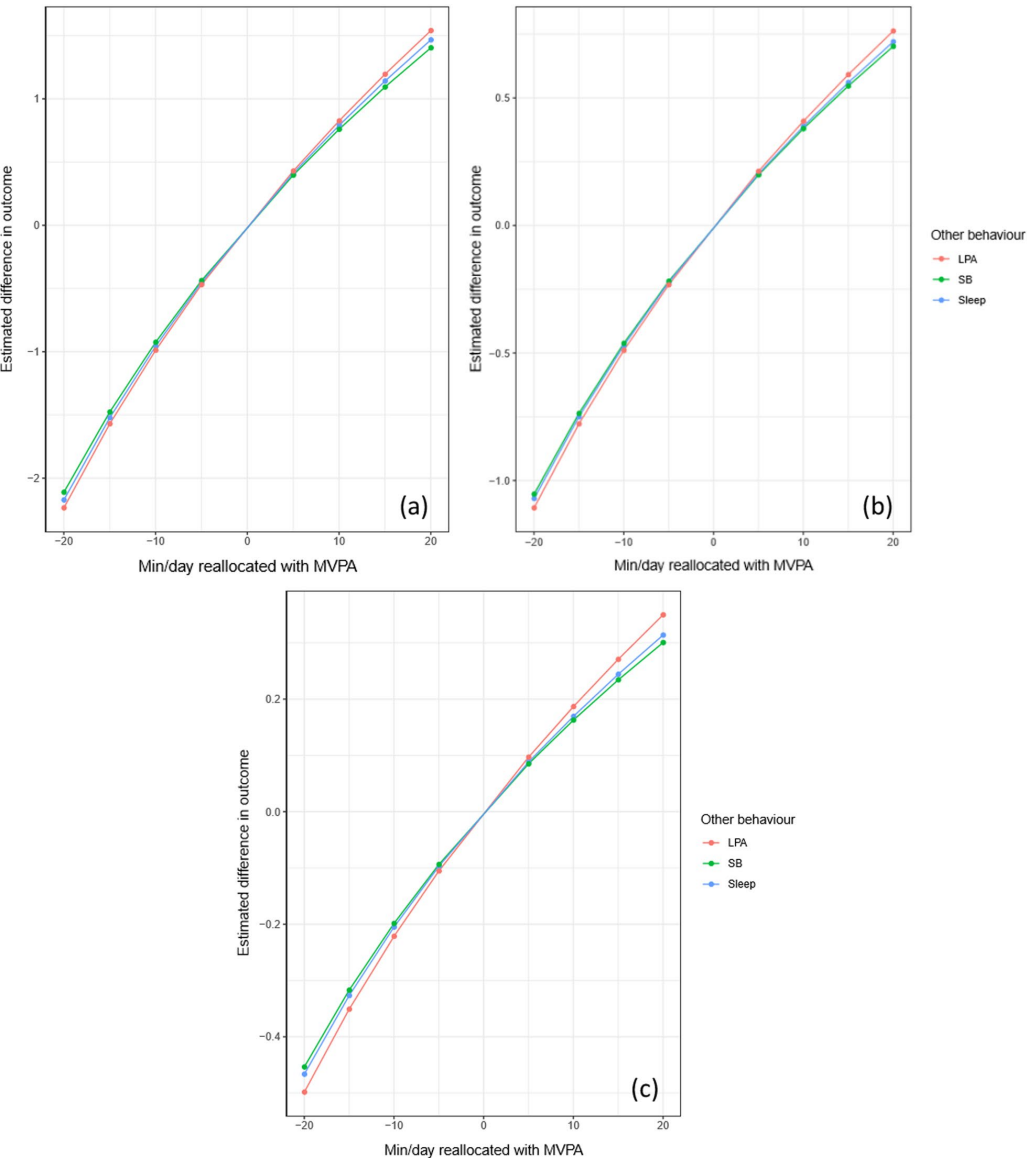
**a**–**c** Estimated difference in Dragon Challenge scores associated with hypothetical time reallocation between pairs of behaviours: difference in all participants’ **a** overall, **b** process and **c** time scores associated with the difference in MVPA to each of the remaining behaviours. For example, adding 20 min to MVPA at the expense of LPA (red line) while keeping sleep and ST constant was associated with an estimated increase of 1.54 in overall score (**a**). y-axis for (**a**) shows unit increases/decreases in overall score, y-axis for (**b**) shows unit increases/decreases in process score, and y-axis for (**c**) shows unit increases/decreases in time score. *MVPA* moderate-to-vigorous physical activity; *LPA* light physical activity; *SB* sedentary time/behaviour

Starting with the average composition of durations for each activity (Table 1) and using compositional isotemporal substitution, Fig. 1a–c and Additional file 2: S2 and Additional file 3: S3 present estimated differences in DC scores when incremental durations of time were added/subtracted from the most influential activity behaviour (Table 3), and reallocated from/to one other activity, keeping all remaining activities constant. For all participants, time reallocations to MVPA from any of the other three behaviours was associated with higher overall, process, and time scores [ES ranged from 0.05 to 0.07 (5 min) and 0.19 to 0.27 (20 min)] (Fig. 1a–c, Additional file 2: S2). Among primary school participants there were positive estimated differences in time score when MVPA hypothetically replaced any other behaviour [ES = 0.08 (5 min) to 0.30 (20 min)]. In secondary school participants, MVPA replacing any other behaviour was associated with positive estimated differences in overall and process scores [ES ranged from 0.08 to 0.10 (5 min) and 0.28 to 0.35 (20 min)], and negative differences in product score were found when sleep hypothetically replaced ST, LPA, and MVPA [ES = 0.06 (5 min) to 0.29 (20 min)] (Additional file 2: S2, Additional file 3: S3).

The sex-stratified by school type compositional isotemporal substitutions showed that (Additional file 2: S2, Additional file 3: S3), for girls, time reallocations to sleep from either ST, LPA, or MVPA was associated with poorer DC scores [ES ranged from 0.03 to 0.10 (5 min) and 0.13 to 0.43 (20 min)] and favourable estimated differences in DC scores were found when MVPA or LPA hypothetically replaced any other activity behaviour [ES ranged from 0.06 to 0.14 (5 min) and 0.23 to 0.63 (20 min)]. For boys, replacing sleep, ST, or LPA with MVPA, was associated with positive estimated differences in DC scores [ES ranged from 0.07 to 0.10 (5 min) and 0.26 to 0.39 (20 min)] and when LPA hypothetically replaced sleep, ST, and MVPA unfavourable estimated differences in DC scores [ES ranged from 0.07 to 0.11 (5 min) and 0.31 to 0.49 (20 min)] were observed (Additional file 4: S4).

## Discussion

In all children and adolescents, we found that activity composition was significantly associated with DC overall, process, and time scores. Among primary school participants, activity composition was associated with time score only, while in secondary school participants it was associated with overall, process, and product scores. Overall, relative to other activity behaviours, MVPA had the greatest association with motor competence outcomes. The largest positive estimated differences in motor competence outcomes occurred when MVPA hypothetically replaced LPA.

Congruent with previous systematic review evidence supporting positive isolated associations between PA levels and motor competence [2, 7], these findings reinforce the key role of MVPA for children’s and adolescents’ motor competence. They also support previous compositional studies examining associations between activity behaviours and motor competence in preschool and primary school children [10, 11]. These showed that reallocating time to MVPA, elicited the largest positive estimated difference in overall motor competence scores [10, 11]. However, contrary to our findings, another compositional study found that relocating time to LPA or sleep, at the expense of ST, was associated with positive differences in motor competence in primary school children from a low socioeconomic status area [12]. Nevertheless, despite one recent systematic review revealing that most previous studies did not find a significant prospective association between PA and subsequent motor competence [3], our findings, as well as others [10, 11] provide cross-sectional evidence that MVPA, relative to other behaviours is associated with motor competence outcomes. Potential reasons for the difference between our findings and studies included within the systematic review evidence [3], are that there were differences in the statistical approach (i.e., time-use compositional analysis), differences in the study measures within our study (e.g., a hybrid measure of motor competence, wrist-worn devices), differences in statistical power, or some combination of these factors.

Among girls, reallocating time to sleep was associated with negative estimated differences and increasing MVPA or LPA was associated with positive differences in DC scores. These findings may reflect that, while sleep plays an important contribution towards healthy 24-h activity behaviours [28], more time spent in sleep is likely not to play a key role in developing motor competence given that it does not provide appropriately challenging opportunities to practice and develop movement skill competency [2–5, 8]. For boys, reallocating time spent to MVPA was associated with positive estimated differences, whereas, increasing time in LPA was associated with negative differences in DC scores. These negative estimated differences may be due to the types of activities that are associated with LPA (e.g., slow walking) and only more intense activities association with MVPA may benefit the development of motor competence [7, 10]. Overall, MVPA had the greatest associations with motor competence outcomes in both boys and girls across school type. The largest positive and negative estimated differences in motor competence outcomes occurred, when MVPA hypothetically replaced LPA or sleep and when LPA or sleep hypothetically replaced MVPA, respectively. Again, consistent with previous systematic reviews showing various associations between PA and motor competence in boys and girls [2, 7], these findings further emphasise the key role of MVPA for both sexes’ motor competence. Notably, no other compositional analysis study [10–13] has investigated sex-stratified associations between activity composition and motor competence outcomes. Thus, we provide new evidence for the contention that time spent in MVPA (relative to other activities) is positively associated with motor competence outcomes irrespective of sex, which could guide the focus of motor competence interventions. For example, practical approaches to increase MVPA are advocated (e.g., active classroom breaks, physically active learning, outdoor play, high quality PE, structured sport/exercise, family activities) [28, 29].

Using a hybrid-based assessment [1, 6], our findings represent a holistic view on the association between activity behaviours and motor competence [1]. The largest estimated increases in overall, process, and time scores were observed when MVPA hypothetically replaced LPA or sleep, whilst the largest estimated decreases occurred when LPA hypothetically replaced MVPA. Further, the largest estimated increases in product score were observed when LPA hypothetically replaced MVPA, and greatest decreases were when sleep or LPA hypothetically replaced MVPA. Previous research utilising compositional data analysis has only considered motor competence outcomes using a process-based approach [10–13], and whilst positive differences in motor competence were found [10–13], the current study provides further evidence that associations exist between MVPA (relative to other activity behaviours) and both process- and product-based motor competence outcomes. Thus, irrespective of a process- or product-based assessment approach used to assess motor competence, our findings suggest that enabling engagement in MVPA is beneficial for motor competence in all children and adolescents.

Interestingly, the estimated differences in motor competence outcomes were greatest when MVPA was hypothetically replaced by sleep or LPA, rather than when MVPA hypothetically replaced these behaviours. These asymmetrical associated differences in physical outcomes involving MVPA have previously been observed in youth studies considering adiposity and fitness [30]. Furthermore, some studies have reported negative associations between ST and motor competence [7, 31]. We found that ST, relative to the other activity behaviours was not significantly associated with any of the motor competence outcomes. However, in Table 2, a negative association was present for the full sample, but inconsistent in the sub-group analyses, therefore, the lack of consistent significant association may be a combination of low statistical power and potential misclassification of LPA as ST. Nonetheless, further studies on the potential negative effects of ST on the development of motor competence are warranted.

In line with the recent review evidence reporting that most previous studies did not find a significant prospective association between PA and subsequent motor competence [3], it is noteworthy, that there were no significant associations observed between the activity compositions and numerous DC scores (Table 2). These non-significant associations between activity behaviours and outcomes require further investigation, but it may mean that other associated factors better estimate motor competence outcomes [3, 4, 8]. Therefore, children/adolescents may benefit from other interventions such as appropriately challenging opportunities, instruction, and feedback during activities, to complement increases in MVPA, as opposed to just time reallocated to MVPA [2–5, 8].

Strengths of this study included wrist-worn device-based assessment of 24-h activity behaviours and use of compositional data analysis to examine how the full activity composition related to motor competence outcomes. Moreover, this is the first compositional analysis study to investigate motor competence outcomes, measured using a hybrid-based assessment that evaluates proficiency in fundamental, combined, and complex movement skills, with a large enough sample size to stratify the data by sex and school type.

However, the study is not without limitations. The cross-sectional design precludes any claims of causal inferences and directionality between the activity composition and motor competence outcomes. There is also longitudinal evidence for a motor competence to subsequent PA association, thus there is a possibility of reverse causality or bi-directional associations [2, 3, 5]. We also had an imbalanced sample of primary and secondary school participants, who were relatively homogenous in terms of area-level socioeconomic status, which limits generalisability. Therefore, future studies should extend this work across the full spectrum of area-level socioeconomic status/neighbourhood deprivation. Finally, although the activity behaviours were defined using validated wrist-worn acceleration cut-points for LPA and MVPA, these reflect absolute intensity rather than relative intensity for each participant (intensity related to individual energy expenditure instead of absolute energy expenditures, and consideration is made towards age-, sex-, and fitness-related differences in the intensities of effort during PA [32]), and therefore, may have resulted in some misclassification of activity behaviours. Cut-point free accelerometer metrics, or machine learning to label activity behaviours and intensities, could be used in future studies to potentially address this issue.

## Conclusion

Overall, the activity behaviour compositions were associated with some but not all motor competence outcomes. Among both sexes, relative to other activity behaviours, MVPA had the greatest associations overall on outcomes. When time was hypothetically reallocated, the largest positive estimated differences in outcomes occurred when MVPA hypothetically replaced LPA or sleep. Therefore, our findings reinforce the key role of MVPA for children and adolescents’ motor competence and highlight the value of compositional data analysis for understanding the collective association of activity behaviours on motor competence (using a hybrid-based approach), which could guide the focus of motor competence interventions. Future studies should investigate longitudinal associations between activity behaviours and motor competence in population-representative samples (Additional file 5: S5).

## Supplementary Information

**Additional file 1.** ILR regression models: which displays compositional isometric log-ratio multiple regression models.

**Additional file 2.** Time reallocations: which displays estimated differences in significant motor competence outcome from time reallocations.

**Additional file 3.** One for one time reallocations: which presents figures showing estimated difference in Dragon Challenge scores associated with time reallocation between pairs of behaviours.

**Additional file 4.** Variation matrices: presents compositional variation matrices of time spent in sleep, ST, LPA, and MVPA.

**Additional file 5.** STROBE checklist: presents completed STROBE checklist for cross-sectional studies.

## Data Availability

The datasets used and/or analysed during the current study are available from the corresponding author on reasonable request.

## Abbreviations

PA: Physical activity
VIRTUE: Viable Integrative Research in Time-Use Epidemiology
IMD: Indices of Multiple Deprivation
BMI: Body mass index
IOTF: International Obesity Task Force
ENMO: Euclidean Norm Minus One
LPA: Light physical activity
MVPA: Moderate-to-vigorous physical activity
ST: Sedentary time
DC: Dragon Challenge
ILR: Isometric log-ratio
ES: Effect size
