# Supplementary material for: Cross-sectional associations between 24-hour activity behaviours and motor competence in youth: a compositional data analysis
Source: J Act Sedentary Sleep Behav. 2022 Sep 1;1:3. doi: 10.1186/s44167-022-00003-3 (PMC11934481; doi:10.1186/s44167-022-00003-3)
Supplement: Supplementary file 1 — Additional file 1. ILR regression models: which displays compositional isometric log-ratio multiple regression models. [file 44167_2022_3_MOESM1_ESM.docx]

Additional Material S1. Compositional isometric log ratio multiple regression models for all participants, primary school participants, and secondary school participants and stratified by sex.

| **All Schools/Participants** | 𝛸^2^ | df | Pr (>𝛸^2^) |
| --- | --- | --- | --- |
| DC Overall Score |  |  |  |
| ILR coordinates | 17.20 | 3 | <0.001*** |
| Age | 11.01 | 1 | <0.001*** |
| Sex | 3.61 | 1 | 0.06 |
| IMD decile | 4.94 | 1 | 0.03* |
| BMI z-score | 14.71 | 1 | <0.001*** |
|  |  |  |  |
| DC Process Score |  |  |  |
| ILR coordinates | 23.79 | 3 | <0.001*** |
| Age | 10.71 | 1 | 0.001** |
| Sex | 4.22 | 1 | 0.04* |
| IMD decile | 5.15 | 1 | 0.023* |
| BMI z-score | 18.07 | 1 | <0.001*** |
|  |  |  |  |
| DC Product Score |  |  |  |
| ILR coordinates | 6.19 | 3 | 0.10 |
| Age | 5.49 | 1 | 0.02* |
| Sex | 1.34 | 1 | 0.25 |
| IMD decile | 2.86 | 1 | 0.09 |
| BMI z-score | 8.81 | 1 | 0.003** |
|  |  |  |  |
| DC Time Score |  |  |  |
| ILR coordinates | 8.87 | 3 | 0.03* |
| Age | 9.57 | 1 | 0.002** |
| Sex | 2.61 | 1 | 0.11 |
| IMD decile | 2.12 | 1 | 0.15 |
| BMI z-score | 3.40 | 1 | 0.07 |
|  |  |  |  |
| **Primary Schools (All)** | 𝛸^2^ | df | Pr (>𝛸^2^) |
| DC Overall Score |  |  |  |
| ILR coordinates | 4.21 | 3 | 0.24 |
| Age | 7.30 | 1 | 0.007** |
| Sex | 1.55 | 1 | 0.21 |
| IMD decile | 0.09 | 1 | 0.76 |
| BMI z-score | 10.41 | 1 | 0.001** |
|  |  |  |  |
| DC Process Score |  |  |  |
| ILR coordinates | 3.74 | 3 | 0.29 |
| Age | 5.41 | 1 | 0.02* |
| Sex | 2.79 | 1 | 0.09 |
| IMD decile | 0.99 | 1 | 0.32 |
| BMI z-score | 14.92 | 1 | <0.001*** |
|  |  |  |  |
| DC Product Score |  |  |  |
| ILR coordinates | 0.90 | 3 | 0.83 |
| Age | 5.14 | 1 | 0.02* |
| Sex | 0.74 | 1 | 0.39 |
| IMD decile | 0.0001 | 1 | 0.99 |
| BMI z-score | 5.95 | 1 | 0.01* |
|  |  |  |  |
| DC Time Score |  |  |  |
| ILR coordinates | 10.84 | 3 | 0.01* |
| Age | 4.77 | 1 | 0.03* |
| Sex | 0.19 | 1 | 0.66 |
| IMD decile | 0.04 | 1 | 0.84 |
| BMI z-score | 1.58 | 1 | 0.21 |
|  |  |  |  |
| **Secondary Schools (All)** | 𝛸^2^ | df | Pr (>𝛸^2^) |
| DC Overall Score |  |  |  |
| ILR coordinates | 17.59 | 3 | <0.001*** |
| Age | 3.89 | 1 | 0.048* |
| Sex | 4.47 | 1 | 0.03* |
| IMD decile | 6.42 | 1 | 0.01* |
| BMI z-score | 5.36 | 1 | 0.02* |
|  |  |  |  |
| DC Process Score |  |  |  |
| ILR coordinates | 23.00 | 3 | <0.001*** |
| Age | 1.50 | 1 | 0.22 |
| Sex | 2.47 | 1 | 0.12 |
| IMD decile | 3.91 | 1 | 0.048* |
| BMI z-score | 4.675 | 1 | 0.03* |
|  |  |  |  |
| DC Product Score |  |  |  |
| ILR coordinates | 9.38 | 3 | 0.02* |
| Age | 3.41 | 1 | 0.06 |
| Sex | 2.04 | 1 | 0.15 |
| IMD decile | 3.65 | 1 | 0.06 |
| BMI z-score | 3.73 | 1 | 0.05 |
|  |  |  |  |
| DC Time Score |  |  |  |
| ILR coordinates | 5.31 | 3 | 0.15 |
| Age | 3.41 | 1 | 0.06 |
| Sex | 7.07 | 1 | 0.008** |
| IMD decile | 6.87 | 1 | 0.009** |
| BMI z-score | 1.75 | 1 | 0.19 |
|  |  |  |  |
| **All Schools (Girls)** | 𝛸^2^ | df | Pr (>𝛸^2^) |
| DC Overall Score |  |  |  |
| ILR coordinates | 13.66 | 3 | 0.003** |
| Age | 4.24 | 1 | 0.04* |
| IMD decile | 10.68 | 1 | 0.001** |
| BMI z-score | 6.15 | 1 | 0.01* |
|  |  |  |  |
| DC Process Score |  |  |  |
| ILR coordinates | 15.46 | 3 | 0.001** |
| Age | 4.02 | 1 | 0.04* |
| IMD decile | 9.35 | 1 | 0.002** |
| BMI z-score | 8.57 | 1 | 0.003** |
|  |  |  |  |
| DC Product Score |  |  |  |
| ILR coordinates | 9.51 | 3 | 0.02* |
| Age | 0.75 | 1 | 0.39 |
| IMD decile | 5.21 | 1 | 0.02* |
| BMI z-score | 3.93 | 1 | 0.047* |
|  |  |  |  |
| DC Time Score |  |  |  |
| ILR coordinates | 2.04 | 3 | 0.56 |
| Age | 4.28 | 1 | 0.04* |
| IMD decile | 8.15 | 1 | 0.004** |
| BMI z-score | 0.70 | 1 | 0.40 |
|  |  |  |  |
| **Primary Schools (Girls)** | 𝛸^2^ | df | Pr (>𝛸^2^) |
| DC Overall Score |  |  |  |
| ILR coordinates | 4.76 | 3 | 0.19 |
| Age | 2.13 | 1 | 0.14 |
| IMD decile | 1.94 | 1 | 0.16 |
| BMI z-score | 7.11 | 1 | 0.008** |
|  |  |  |  |
| DC Process Score |  |  |  |
| ILR coordinates | 3.30 | 3 | 0.35 |
| Age | 0.17 | 1 | 0.68 |
| IMD decile | 2.46 | 1 | 0.12 |
| BMI z-score | 11.26 | 1 | <0.001*** |
|  |  |  |  |
| DC Product Score |  |  |  |
| ILR coordinates | 9.56 | 3 | 0.02* |
| Age | 4.73 | 1 | 0.03* |
| IMD decile | 0.26 | 1 | 0.61 |
| BMI z-score | 5.33 | 1 | 0.02* |
|  |  |  |  |
| DC Time Score |  |  |  |
| ILR coordinates | 4.26 | 3 | 0.23 |
| Age | 1.08 | 1 | 0.30 |
| IMD decile | 5.74 | 1 | 0.02* |
| BMI z-score | 0.40 | 1 | 0.53 |
|  |  |  |  |
| **Secondary Schools (Girls)** | 𝛸^2^ | df | Pr (>𝛸^2^) |
| DC Overall Score |  |  |  |
| ILR coordinates | 11.86 | 3 | 0.008** |
| Age | 5.42 | 1 | 0.02* |
| IMD decile | 5.39 | 1 | 0.02* |
| BMI z-score | 1.42 | 1 | 0.23 |
|  |  |  |  |
| DC Process Score |  |  |  |
| ILR coordinates | 15.38 | 3 | 0.002** |
| Age | 2.96 | 1 | 0.09 |
| IMD decile | 3.67 | 1 | 0.06 |
| BMI z-score | 1.24 | 1 | 0.27 |
|  |  |  |  |
| DC Product Score |  |  |  |
| ILR coordinates | 8.93 | 3 | 0.03* |
| Age | 4.43 | 1 | 0.04* |
| IMD decile | 2.90 | 1 | 0.09 |
| BMI z-score | 1.30 | 1 | 0.26 |
|  |  |  |  |
| DC Time Score |  |  |  |
| ILR coordinates | 1.06 | 3 | 0.79 |
| Age | 3.49 | 1 | 0.06 |
| IMD decile | 5.33 | 1 | 0.02* |
| BMI z-score | 0.18 | 1 | 0.67 |
|  |  |  |  |
| **All Schools (Boys)** | 𝛸^2^ | df | Pr (>𝛸^2^) |
| DC Overall Score |  |  |  |
| ILR coordinates | 12.94 | 3 | 0.005** |
| Age | 10.00 | 1 | 0.002** |
| IMD decile | 0.03 | 1 | 0.87 |
| BMI z-score | 9.80 | 1 | 0.002** |
|  |  |  |  |
| DC Process Score |  |  |  |
| ILR coordinates | 14.57 | 3 | 0.002** |
| Age | 10.70 | 1 | 0.001** |
| IMD decile | 0.04 | 1 | 0.83 |
| BMI z-score | 10.51 | 1 | 0.001** |
|  |  |  |  |
| DC Product Score |  |  |  |
| ILR coordinates | 6.25 | 3 | 0.10 |
| Age | 4.21 | 1 | 0.04* |
| IMD decile | 0.15 | 1 | 0.70 |
| BMI z-score | 5.17 | 1 | 0.02* |
|  |  |  |  |
| DC Time Score |  |  |  |
| ILR coordinates | 8.01 | 3 | 0.045* |
| Age | 9.48 | 1 | 0.002** |
| IMD decile | 0.29 | 1 | 0.59 |
| BMI z-score | 4.00 | 1 | 0.045* |
|  |  |  |  |
| **Primary Schools (Boys)** | 𝛸^2^ | df | Pr (>𝛸^2^) |
| DC Overall Score |  |  |  |
| ILR coordinates | 11.69 | 3 | 0.009** |
| Age | 7.19 | 1 | 0.007** |
| IMD decile | 1.52 | 1 | 0.22 |
| BMI z-score | 4.90 | 1 | 0.03* |
|  |  |  |  |
| DC Process Score |  |  |  |
| ILR coordinates | 8.72 | 3 | 0.03* |
| Age | 8.37 | 1 | 0.004** |
| IMD decile | 0.27 | 1 | 0.60 |
| BMI z-score | 5.18 | 1 | 0.02* |
|  |  |  |  |
| DC Product Score |  |  |  |
| ILR coordinates | 8.16 | 3 | 0.04* |
| Age | 2.52 | 1 | 0.11 |
| IMD decile | 0.53 | 1 | 0.47 |
| BMI z-score | 2.62 | 1 | 0.11 |
|  |  |  |  |
| DC Time Score |  |  |  |
| ILR coordinates | 10.52 | 3 | 0.01* |
| Age | 6.72 | 1 | 0.0095** |
| IMD decile | 5.37 | 1 | 0.02* |
| BMI z-score | 1.40 | 1 | 0.24 |
|  |  |  |  |
| **Secondary Schools (Boys)** | 𝛸^2^ | df | Pr (>𝛸^2^) |
| DC Overall Score |  |  |  |
| ILR coordinates | 7.33 | 3 | 0.06 |
| Age | 0.39 | 1 | 0.53 |
| IMD decile | 1.34 | 1 | 0.25 |
| BMI z-score | 5.63 | 1 | 0.02* |
|  |  |  |  |
| DC Process Score |  |  |  |
| ILR coordinates | 8.66 | 3 | 0.03* |
| Age | 0.21 | 1 | 0.64 |
| IMD decile | 0.37 | 1 | 0.54 |
| BMI z-score | 5.90 | 1 | 0.01* |
|  |  |  |  |
| DC Product Score |  |  |  |
| ILR coordinates | 2.97 | 3 | 0.40 |
| Age | 0.61 | 1 | 0.44 |
| IMD decile | 1.06 | 1 | 0.30 |
| BMI z-score | 2.83 | 1 | 0.09 |
|  |  |  |  |
| DC Time Score |  |  |  |
| ILR coordinates | 5.82 | 3 | 0.12 |
| Age | 0.38 | 1 | 0.54 |
| IMD decile | 1.94 | 1 | 0.16 |
| BMI z-score | 2.30 | 1 | 0.13 |
|  |  |  |  |
| Note. DC – Dragon Challenge; ILR – Isometric Log Ratio; IMD – Indices of Multiple Deprivation; BMI – Body Mass Index. | | | |
