# Supplementary material for: Cross-sectional associations between 24-hour activity behaviours and motor competence in youth: a compositional data analysis
Source: J Act Sedentary Sleep Behav. 2022 Sep 1;1:3. doi: 10.1186/s44167-022-00003-3 (PMC11934481; doi:10.1186/s44167-022-00003-3)
Supplement: Supplementary file 2 — Additional file 2. Time reallocations: which displays estimated differences in significant motor competence outcome from time reallocations. [file 44167_2022_3_MOESM2_ESM.docx]

Additional Material S2. Time reallocations for motor competence outcomes that were significantly associated with the activity compositions and the activity behaviour isometric log-ratio pivot coordinates

| **All Schools/Participants** | Estimated difference in Dragon Challenge Overall Score | | | | | |
| --- | --- | --- | --- | --- | --- | --- |
| Time reallocations | Increase MVPA  Decrease Sleep | Increase MVPA  Decrease ST | Increase MVPA  Decrease LPA | Decrease MVPA  Increase Sleep | Decrease MVPA  Increase ST | Decrease MVPA  Increase LPA |
| 20 min | 1.47 | 1.40 | 1.54 | -2.17 | -2.11 | -2.23 |
| ES | 0.22 | 0.21 | 0.23 | -0.33 | -0.32 | -0.34 |
| 15 min | 1.14 | 1.09 | 1.19 | -1.52 | -1.48 | -1.57 |
| ES | 0.17 | 0.17 | 0.18 | -0.23 | -0.22 | -0.24 |
| 10 min | 0.79 | 0.76 | 0.83 | -0.96 | -0.93 | -0.99 |
| ES | 0.12 | 0.12 | 0.13 | -0.14 | -0.14 | -0.15 |
| 5 min | 0.41 | 0.40 | 0.43 | -0.45 | -0.44 | -0.47 |
| ES | 0.06 | 0.06 | 0.07 | -0.07 | -0.07 | -0.07 |
| Note. ES – Effect Size; ST – Sedentary Time; LPA – Light Physical Activity; MVPA – Moderate-to-Vigorous Physical Activity | | | | | | |

| **All Schools/Participants** | Estimated difference in Dragon Challenge Process Score | | | | | |
| --- | --- | --- | --- | --- | --- | --- |
| Time reallocations | Increase MVPA  Decrease Sleep | Increase MVPA  Decrease ST | Increase MVPA  Decrease LPA | Decrease MVPA  Increase Sleep | Decrease MVPA  Increase ST | Decrease MVPA  Increase LPA |
| 20 min | 0.72 | 0.70 | 0.76 | -1.07 | -1.05 | -1.11 |
| ES | 0.25 | 0.25 | 0.27 | -0.38 | -0.37 | -0.39 |
| 15 min | 0.56 | 0.55 | 0.59 | -0.75 | -0.74 | -0.78 |
| ES | 0.20 | 0.19 | 0.21 | -0.26 | -0.26 | -0.27 |
| 10 min | 0.39 | 0.38 | 0.41 | -0.47 | -0.46 | -0.49 |
| ES | 0.14 | 0.13 | 0.14 | -0.17 | -0.16 | -0.17 |
| 5 min | 0.20 | 0.20 | 0.21 | -0.22 | -0.22 | -0.23 |
| ES | 0.07 | 0.07 | 0.07 | -0.08 | -0.08 | -0.08 |
| Note. ES – Effect Size; ST – Sedentary Time; LPA – Light Physical Activity; MVPA – Moderate-to-Vigorous Physical Activity | | | | | | |

| **All Schools/Participants** | Estimated difference in Dragon Challenge Time Score | | | | | |
| --- | --- | --- | --- | --- | --- | --- |
| Time reallocations | Increase MVPA  Decrease Sleep | Increase MVPA  Decrease ST | Increase MVPA  Decrease LPA | Decrease MVPA  Increase Sleep | Decrease MVPA  Increase ST | Decrease MVPA  Increase LPA |
| 20 min | 0.31 | 0.30 | 0.35 | -0.47 | -0.45 | -0.50 |
| ES | 0.17 | 0.16 | 0.19 | -0.25 | -0.24 | -0.27 |
| 15 min | 0.24 | 0.23 | 0.27 | -0.33 | -0.32 | -0.35 |
| ES | 0.13 | 0.13 | 0.14 | -0.17 | -0.17 | -0.19 |
| 10 min | 0.17 | 0.16 | 0.19 | -0.21 | -0.20 | -0.22 |
| ES | 0.09 | 0.09 | 0.10 | -0.11 | -0.11 | -0.12 |
| 5 min | 0.09 | 0.08 | 0.10 | -0.10 | -0.09 | -0.11 |
| ES | 0.05 | 0.05 | 0.05 | -0.05 | -0.05 | -0.06 |
| Note. ES – Effect Size; ST – Sedentary Time; LPA – Light Physical Activity; MVPA – Moderate-to-Vigorous Physical Activity | | | | | | |

| **Primary Schools (All)** | Estimated difference in Dragon Challenge Time Score | | | | | |
| --- | --- | --- | --- | --- | --- | --- |
| Time reallocations | Increase MVPA  Decrease Sleep | Increase MVPA  Decrease ST | Increase MVPA  Decrease LPA | Decrease MVPA  Increase Sleep | Decrease MVPA  Increase ST | Decrease MVPA  Increase LPA |
| 20 min | 0.40 | 0.49 | 0.59 | -0.59 | -0.68 | -0.77 |
| ES | 0.20 | 0.25 | 0.30 | -0.30 | -0.35 | -0.39 |
| 15 min | 0.31 | 0.38 | 0.46 | -0.42 | -0.48 | -0.55 |
| ES | 0.16 | 0.19 | 0.23 | -0.21 | -0.25 | -0.28 |
| 10 min | 0.22 | 0.26 | 0.31 | -0.26 | -0.31 | -0.35 |
| ES | 0.11 | 0.13 | 0.16 | -0.13 | -0.16 | -0.18 |
| 5 min | 0.11 | 0.14 | 0.16 | -0.12 | -0.15 | -0.17 |
| ES | 0.06 | 0.07 | 0.08 | -0.06 | -0.07 | -0.09 |
| Note. ES – Effect Size; ST – Sedentary Time; LPA – Light Physical Activity; MVPA – Moderate-to-Vigorous Physical Activity | | | | | | |

| **Secondary Schools (All)** | Estimated difference in Dragon Challenge Overall Score | | | | | |
| --- | --- | --- | --- | --- | --- | --- |
| Time reallocations | Increase MVPA  Decrease Sleep | Increase MVPA  Decrease ST | Increase MVPA  Decrease LPA | Decrease MVPA  Increase Sleep | Decrease MVPA  Increase ST | Decrease MVPA  Increase LPA |
| 20 min | 1.81 | 1.55 | 1.27 | -2.83 | -2.59 | -2.34 |
| ES | 0.28 | 0.24 | 0.19 | -0.43 | -0.39 | -0.36 |
| 15 min | 1.41 | 1.22 | 1.01 | -1.95 | -1.77 | -1.57 |
| ES | 0.21 | 0.19 | 0.15 | -0.30 | -0.27 | -0.24 |
| 10 min | 0.98 | 0.85 | 0.72 | -1.21 | -1.09 | -0.96 |
| ES | 0.15 | 0.13 | 0.11 | -0.18 | -0.17 | -0.15 |
| 5 min | 0.51 | 0.45 | 0.38 | -0.57 | -0.51 | -0.44 |
| ES | 0.08 | 0.07 | 0.06 | -0.09 | -0.08 | -0.07 |
| Note. ES – Effect Size; ST – Sedentary Time; LPA – Light Physical Activity; MVPA – Moderate-to-Vigorous Physical Activity | | | | | | |

| **Secondary Schools (All)** | Estimated difference in Dragon Challenge Process Score | | | | | |
| --- | --- | --- | --- | --- | --- | --- |
| Time reallocations | Increase MVPA  Decrease Sleep | Increase MVPA  Decrease ST | Increase MVPA  Decrease LPA | Decrease MVPA  Increase Sleep | Decrease MVPA  Increase ST | Decrease MVPA  Increase LPA |
| 20 min | 1.00 | 0.92 | 0.87 | -1.61 | -1.53 | -1.49 |
| ES | 0.35 | 0.32 | 0.30 | -0.56 | -0.53 | -0.51 |
| 15 min | 0.78 | 0.72 | 0.68 | -1.11 | -1.05 | -1.01 |
| ES | 0.27 | 0.25 | 0.24 | -0.38 | -0.36 | -0.35 |
| 10 min | 0.55 | 0.51 | 0.48 | -0.68 | -0.64 | -0.62 |
| ES | 0.19 | 0.18 | 0.17 | -0.24 | -0.22 | -0.21 |
| 5 min | 0.29 | 0.27 | 0.25 | -0.32 | -0.30 | -0.29 |
| ES | 0.10 | 0.09 | 0.09 | -0.11 | -0.10 | -0.10 |
| Note. ES – Effect Size; ST – Sedentary Time; LPA – Light Physical Activity; MVPA – Moderate-to-Vigorous Physical Activity | | | | | | |

| **Secondary Schools (All)** | Estimated difference in Dragon Challenge Product Score | | | | | |
| --- | --- | --- | --- | --- | --- | --- |
| Time reallocations | Increase Sleep  Decrease ST | Increase Sleep  Decrease LPA | Increase Sleep  Decrease MVPA | Decrease Sleep  Increase ST | Decrease Sleep  Increase LPA | Decrease Sleep  Increase MVPA |
| 20 min | -0.11 | -0.24 | -1.01 | 0.11 | 0.23 | 0.65 |
| ES | -0.03 | -0.07 | -0.29 | 0.03 | 0.07 | 0.19 |
| 15 min | -0.08 | -0.18 | -0.69 | 0.09 | 0.17 | 0.51 |
| ES | -0.02 | -0.05 | -0.20 | 0.03 | 0.05 | 0.15 |
| 10 min | -0.06 | -0.12 | -0.43 | 0.06 | 0.12 | 0.35 |
| ES | -0.02 | -0.03 | -0.13 | 0.02 | 0.03 | 0.10 |
| 5 min | -0.03 | -0.06 | -0.20 | 0.03 | 0.06 | 0.18 |
| ES | -0.01 | -0.02 | -0.06 | 0.01 | 0.02 | 0.05 |
| Note. ES – Effect Size; ST – Sedentary Time; LPA – Light Physical Activity; MVPA – Moderate-to-Vigorous Physical Activity | | | | | | |

| **All Schools (Girls)** | Estimated difference in Dragon Challenge Overall Score | | | | | |
| --- | --- | --- | --- | --- | --- | --- |
| Time reallocations | Increase Sleep  Decrease ST | Increase Sleep  Decrease LPA | Increase Sleep  Decrease MVPA | Decrease Sleep  Increase ST | Decrease Sleep  Increase LPA | Decrease Sleep  Increase MVPA |
| 20 min | -0.26 | -0.76 | -1.59 | 0.27 | 0.72 | 1.10 |
| ES | -0.04 | -0.12 | -0.24 | 0.04 | 0.11 | 0.17 |
| 15 min | -0.19 | -0.57 | -1.11 | 0.20 | 0.55 | 0.85 |
| ES | -0.03 | -0.09 | -0.17 | 0.03 | 0.08 | 0.13 |
| 10 min | -0.13 | -0.37 | -0.70 | 0.13 | 0.37 | 0.59 |
| ES | -0.02 | -0.06 | -0.11 | 0.02 | 0.06 | 0.09 |
| 5 min | -0.07 | -0.19 | -0.33 | 0.07 | 0.18 | 0.30 |
| ES | -0.01 | -0.03 | -0.05 | 0.01 | 0.03 | 0.05 |
| Note. ES – Effect Size; ST – Sedentary Time; LPA – Light Physical Activity; MVPA – Moderate-to-Vigorous Physical Activity | | | | | | |

| **All Schools (Girls)** | Estimated difference in Dragon Challenge Process Score | | | | | |
| --- | --- | --- | --- | --- | --- | --- |
| Time reallocations | Increase MVPA  Decrease Sleep | Increase MVPA  Decrease ST | Increase MVPA  Decrease LPA | Decrease MVPA  Increase Sleep | Decrease MVPA  Increase ST | Decrease MVPA  Increase LPA |
| 20 min | 0.66 | 0.55 | 0.41 | -0.99 | -0.88 | -0.75 |
| ES | 0.23 | 0.19 | 0.14 | -0.34 | -0.30 | -0.26 |
| 15 min | 0.52 | 0.43 | 0.32 | -0.69 | -0.61 | -0.51 |
| ES | 0.18 | 0.15 | 0.11 | -0.24 | -0.21 | -0.18 |
| 10 min | 0.36 | 0.30 | 0.23 | -0.43 | -0.38 | -0.31 |
| ES | 0.12 | 0.10 | 0.08 | -0.15 | -0.13 | -0.11 |
| 5 min | 0.19 | 0.16 | 0.12 | -0.20 | -0.18 | -0.14 |
| ES | 0.06 | 0.05 | 0.04 | -0.07 | -0.06 | -0.05 |
| Note. ES – Effect Size; ST – Sedentary Time; LPA – Light Physical Activity; MVPA – Moderate-to-Vigorous Physical Activity | | | | | | |

| **All Schools (Girls)** | Estimated difference in Dragon Challenge Product Score | | | | | |
| --- | --- | --- | --- | --- | --- | --- |
| Time reallocations | Increase Sleep  Decrease ST | Increase Sleep  Decrease LPA | Increase Sleep  Decrease MVPA | Decrease Sleep  Increase ST | Decrease Sleep  Increase LPA | Decrease Sleep  Increase MVPA |
| 20 min | -0.17 | -0.44 | -0.44 | 0.17 | 0.42 | 0.33 |
| ES | -0.05 | -0.13 | -0.13 | 0.05 | 0.12 | 0.10 |
| 15 min | -0.13 | -0.33 | -0.31 | 0.13 | 0.32 | 0.26 |
| ES | -0.04 | -0.10 | -0.09 | 0.04 | 0.09 | 0.08 |
| 10 min | -0.08 | -0.22 | -0.20 | 0.09 | 0.21 | 0.17 |
| ES | -0.02 | -0.06 | -0.06 | 0.03 | 0.06 | 0.05 |
| 5 min | -0.04 | -0.11 | -0.10 | 0.04 | 0.11 | 0.09 |
| ES | -0.01 | -0.03 | -0.03 | 0.01 | 0.03 | 0.03 |
| Note. ES – Effect Size; ST – Sedentary Time; LPA – Light Physical Activity; MVPA – Moderate-to-Vigorous Physical Activity | | | | | | |

| **Primary Schools (Girls)** | Estimated difference in Dragon Challenge Product Score | | | | | |
| --- | --- | --- | --- | --- | --- | --- |
| Time reallocations | Increase LPA  Decrease Sleep | Increase LPA  Decrease ST | Increase LPA  Decrease MVPA | Decrease LPA  Increase Sleep | Decrease LPA  Increase ST | Decrease LPA  Increase MVPA |
| 20 min | 0.78 | 0.55 | 1.98 | -0.84 | -0.61 | -1.59 |
| ES | 0.25 | 0.17 | 0.63 | -0.26 | -0.19 | -0.50 |
| 15 min | 0.59 | 0.42 | 1.42 | -0.62 | -0.45 | -1.21 |
| ES | 0.19 | 0.13 | 0.45 | -0.20 | -0.14 | -0.38 |
| 10 min | 0.40 | 0.28 | 0.92 | -0.41 | -0.30 | -0.82 |
| ES | 0.13 | 0.09 | 0.29 | -0.13 | -0.09 | -0.26 |
| 5 min | 0.20 | 0.14 | 0.44 | -0.20 | -0.15 | -0.42 |
| ES | 0.06 | 0.05 | 0.14 | -0.06 | -0.05 | -0.13 |
| Note. ES – Effect Size; ST – Sedentary Time; LPA – Light Physical Activity; MVPA – Moderate-to-Vigorous Physical Activity | | | | | | |

| **Secondary Schools (Girls)** | Estimated difference in Dragon Challenge Overall Score | | | | | |
| --- | --- | --- | --- | --- | --- | --- |
| Time reallocations | Increase Sleep  Decrease ST | Increase Sleep  Decrease LPA | Increase Sleep  Decrease MVPA | Decrease Sleep  Increase ST | Decrease Sleep  Increase LPA | Decrease Sleep  Increase MVPA |
| 20 min | -0.59 | -1.01 | -3.76 | 0.60 | 0.98 | 2.30 |
| ES | -0.08 | -0.14 | -0.54 | 0.09 | 0.14 | 0.33 |
| 15 min | -0.44 | -0.75 | -2.55 | 0.45 | 0.74 | 1.79 |
| ES | -0.06 | -0.11 | -0.36 | 0.06 | 0.11 | 0.26 |
| 10 min | -0.29 | -0.50 | -1.56 | 0.30 | 0.49 | 1.25 |
| ES | -0.04 | -0.07 | -0.22 | 0.04 | 0.07 | 0.18 |
| 5 min | -0.15 | -0.25 | -0.73 | 0.15 | 0.25 | 0.65 |
| ES | -0.02 | -0.04 | -0.10 | 0.02 | 0.04 | 0.09 |
| Note. ES – Effect Size; ST – Sedentary Time; LPA – Light Physical Activity; MVPA – Moderate-to-Vigorous Physical Activity | | | | | | |

| **Secondary Schools (Girls)** | Estimated difference in Dragon Challenge Process Score | | | | | |
| --- | --- | --- | --- | --- | --- | --- |
| Time reallocations | Increase MVPA  Decrease Sleep | Increase MVPA  Decrease ST | Increase MVPA  Decrease LPA | Decrease MVPA  Increase Sleep | Decrease MVPA  Increase ST | Decrease MVPA  Increase LPA |
| 20 min | 1.30 | 1.08 | 0.95 | -2.20 | -1.99 | -1.87 |
| ES | 0.42 | 0.35 | 0.30 | -0.71 | -0.64 | -0.60 |
| 15 min | 1.02 | 0.85 | 0.75 | -1.48 | -1.32 | -1.23 |
| ES | 0.33 | 0.27 | 0.24 | -0.48 | -0.42 | -0.39 |
| 10 min | 0.71 | 0.60 | 0.53 | -0.91 | -0.80 | -0.74 |
| ES | 0.23 | 0.19 | 0.17 | -0.29 | -0.26 | -0.24 |
| 5 min | 0.37 | 0.32 | 0.29 | -0.42 | -0.37 | -0.34 |
| ES | 0.12 | 0.10 | 0.09 | -0.14 | -0.12 | -0.11 |
| Note. ES – Effect Size; ST – Sedentary Time; LPA – Light Physical Activity; MVPA – Moderate-to-Vigorous Physical Activity | | | | | | |

| **Secondary Schools (Girls)** | Estimated difference in Dragon Challenge Product Score | | | | | |
| --- | --- | --- | --- | --- | --- | --- |
| Time reallocations | Increase Sleep  Decrease ST | Increase Sleep  Decrease LPA | Increase Sleep  Decrease MVPA | Decrease Sleep  Increase ST | Decrease Sleep  Increase LPA | Decrease Sleep  Increase MVPA |
| 20 min | -0.34 | -0.50 | -1.55 | 0.35 | 0.49 | 0.98 |
| ES | -0.09 | -0.14 | -0.43 | 0.10 | 0.14 | 0.27 |
| 15 min | -0.26 | -0.38 | -1.06 | 0.26 | 0.37 | 0.76 |
| ES | -0.07 | -0.10 | -0.29 | 0.07 | 0.10 | 0.21 |
| 10 min | -0.17 | -0.25 | -0.65 | 0.17 | 0.25 | 0.53 |
| ES | -0.05 | -0.07 | -0.18 | 0.05 | 0.07 | 0.15 |
| 5 min | -0.09 | -0.12 | -0.31 | 0.09 | 0.12 | 0.28 |
| ES | -0.02 | -0.03 | -0.09 | 0.02 | 0.03 | 0.08 |
| Note. ES – Effect Size; ST – Sedentary Time; LPA – Light Physical Activity; MVPA – Moderate-to-Vigorous Physical Activity | | | | | | |

| **All Schools (Boys)** | Estimated difference in Dragon Challenge Overall Score | | | | | |
| --- | --- | --- | --- | --- | --- | --- |
| Time reallocations | Increase MVPA  Decrease Sleep | Increase MVPA  Decrease ST | Increase MVPA  Decrease LPA | Decrease MVPA  Increase Sleep | Decrease MVPA  Increase ST | Decrease MVPA  Increase LPA |
| 20 min | 1.80 | 1.83 | 2.45 | -2.59 | -2.62 | -3.19 |
| ES | 0.27 | 0.27 | 0.37 | -0.39 | -0.39 | -0.48 |
| 15 min | 1.40 | 1.42 | 1.88 | -1.83 | -1.85 | -2.28 |
| ES | 0.21 | 0.21 | 0.28 | -0.28 | -0.28 | -0.34 |
| 10 min | 0.97 | 0.98 | 1.29 | -1.16 | -1.17 | -1.46 |
| ES | 0.15 | 0.15 | 0.19 | -0.17 | -0.18 | -0.22 |
| 5 min | 0.50 | 0.51 | 0.66 | -0.55 | -0.56 | -0.70 |
| ES | 0.08 | 0.08 | 0.10 | -0.08 | -0.08 | -0.11 |
| Note. ES – Effect Size; ST – Sedentary Time; LPA – Light Physical Activity; MVPA – Moderate-to-Vigorous Physical Activity | | | | | | |

| **All Schools (Boys)** | Estimated difference in Dragon Challenge Process Score | | | | | |
| --- | --- | --- | --- | --- | --- | --- |
| Time reallocations | Increase MVPA  Decrease Sleep | Increase MVPA  Decrease ST | Increase MVPA  Decrease LPA | Decrease MVPA  Increase Sleep | Decrease MVPA  Increase ST | Decrease MVPA  Increase LPA |
| 20 min | 0.77 | 0.79 | 1.03 | -1.12 | -1.13 | -1.35 |
| ES | 0.28 | 0.29 | 0.37 | -0.40 | -0.41 | -0.49 |
| 15 min | 0.60 | 0.61 | 0.79 | -0.79 | -0.80 | -0.96 |
| ES | 0.22 | 0.22 | 0.29 | -0.29 | -0.29 | -0.35 |
| 10 min | 0.42 | 0.42 | 0.54 | -0.50 | -0.51 | -0.62 |
| ES | 0.15 | 0.15 | 0.20 | -0.18 | -0.18 | -0.22 |
| 5 min | 0.22 | 0.22 | 0.28 | -0.24 | -0.24 | -0.30 |
| ES | 0.08 | 0.08 | 0.10 | -0.09 | -0.09 | -0.11 |
| Note. ES – Effect Size; ST – Sedentary Time; LPA – Light Physical Activity; MVPA – Moderate-to-Vigorous Physical Activity | | | | | | |

| **All Schools (Boys)** | Estimated difference in Dragon Challenge Time Score | | | | | |
| --- | --- | --- | --- | --- | --- | --- |
| Time reallocations | Increase MVPA  Decrease Sleep | Increase MVPA  Decrease ST | Increase MVPA  Decrease LPA | Decrease MVPA  Increase Sleep | Decrease MVPA  Increase ST | Decrease MVPA  Increase LPA |
| 20 min | 0.45 | 0.43 | 0.54 | -0.64 | -0.62 | -0.72 |
| ES | 0.22 | 0.21 | 0.26 | -0.31 | -0.30 | -0.35 |
| 15 min | 0.35 | 0.34 | 0.41 | -0.45 | -0.44 | -0.51 |
| ES | 0.17 | 0.16 | 0.20 | -0.22 | -0.21 | -0.25 |
| 10 min | 0.24 | 0.23 | 0.28 | -0.29 | -0.28 | -0.33 |
| ES | 0.12 | 0.11 | 0.14 | -0.14 | -0.14 | -0.16 |
| 5 min | 0.13 | 0.12 | 0.15 | -0.14 | -0.13 | -0.16 |
| ES | 0.06 | 0.06 | 0.07 | -0.07 | -0.06 | -0.08 |
| Note. ES – Effect Size; ST – Sedentary Time; LPA – Light Physical Activity; MVPA – Moderate-to-Vigorous Physical Activity | | | | | | |

| **Primary Schools (Boys)** | Estimated difference in Dragon Challenge Overall Score | | | | | |
| --- | --- | --- | --- | --- | --- | --- |
| Time reallocations | Increase LPA  Decrease Sleep | Increase LPA  Decrease ST | Increase LPA  Decrease MVPA | Decrease LPA  Increase Sleep | Decrease LPA  Increase ST | Decrease LPA  Increase MVPA |
| 20 min | -1.31 | -0.86 | -3.41 | 1.39 | 0.96 | 2.84 |
| ES | -0.19 | -0.12 | -0.49 | 0.20 | 0.14 | 0.41 |
| 15 min | -0.99 | -0.65 | -2.48 | 1.04 | 0.71 | 2.16 |
| ES | -0.14 | -0.09 | -0.36 | 0.15 | 0.10 | 0.31 |
| 10 min | -0.66 | -0.44 | -1.60 | 0.68 | 0.47 | 1.47 |
| ES | -0.10 | -0.06 | -0.23 | 0.10 | 0.07 | 0.21 |
| 5 min | -0.33 | -0.22 | -0.78 | 0.34 | 0.23 | 0.75 |
| ES | -0.05 | -0.03 | -0.11 | 0.05 | 0.03 | 0.11 |
| Note. ES – Effect Size; ST – Sedentary Time; LPA – Light Physical Activity; MVPA – Moderate-to-Vigorous Physical Activity | | | | | | |

| **Primary Schools (Boys)** | Estimated difference in Dragon Challenge Process Score | | | | | |
| --- | --- | --- | --- | --- | --- | --- |
| Time reallocations | Increase LPA  Decrease Sleep | Increase LPA  Decrease ST | Increase LPA  Decrease MVPA | Decrease LPA  Increase Sleep | Decrease LPA  Increase ST | Decrease LPA  Increase MVPA |
| 20 min | -0.47 | -0.35 | -1.34 | 0.50 | 0.39 | 1.11 |
| ES | -0.16 | -0.12 | -0.46 | 0.17 | 0.13 | 0.38 |
| 15 min | -0.35 | -0.27 | -0.97 | 0.37 | 0.29 | 0.85 |
| ES | -0.12 | -0.09 | -0.34 | 0.13 | 0.10 | 0.29 |
| 10 min | -0.24 | -0.18 | -0.63 | 0.25 | 0.19 | 0.58 |
| ES | -0.08 | -0.06 | -0.22 | 0.08 | 0.07 | 0.20 |
| 5 min | -0.12 | -0.09 | -0.31 | 0.12 | 0.09 | 0.29 |
| ES | -0.04 | -0.03 | -0.11 | 0.04 | 0.03 | 0.10 |
| Note. ES – Effect Size; ST – Sedentary Time; LPA – Light Physical Activity; MVPA – Moderate-to-Vigorous Physical Activity | | | | | | |

| **Primary Schools (Boys)** | Estimated difference in Dragon Challenge Product Score | | | | | |
| --- | --- | --- | --- | --- | --- | --- |
| Time reallocations | Increase LPA  Decrease Sleep | Increase LPA  Decrease ST | Increase LPA  Decrease MVPA | Decrease LPA  Increase Sleep | Decrease LPA  Increase ST | Decrease LPA  Increase MVPA |
| 20 min | -0.58 | -0.33 | -1.02 | 0.61 | 0.38 | 0.89 |
| ES | -0.17 | -0.10 | -0.31 | 0.18 | 0.11 | 0.27 |
| 15 min | -0.44 | -0.25 | -0.74 | 0.45 | 0.28 | 0.67 |
| ES | -0.13 | -0.08 | -0.23 | 0.14 | 0.08 | 0.20 |
| 10 min | -0.29 | -0.17 | -0.49 | 0.30 | 0.18 | 0.45 |
| ES | -0.09 | -0.05 | -0.15 | 0.09 | 0.06 | 0.14 |
| 5 min | -0.15 | -0.09 | -0.24 | 0.15 | 0.09 | 0.23 |
| ES | -0.04 | -0.03 | -0.07 | 0.05 | 0.03 | 0.07 |
| Note. ES – Effect Size; ST – Sedentary Time; LPA – Light Physical Activity; MVPA – Moderate-to-Vigorous Physical Activity | | | | | | |

| **Primary Schools (Boys)** | Estimated difference in Dragon Challenge Time Score | | | | | |
| --- | --- | --- | --- | --- | --- | --- |
| Time reallocations | Increase MVPA  Decrease Sleep | Increase MVPA  Decrease ST | Increase MVPA  Decrease LPA | Decrease MVPA  Increase Sleep | Decrease MVPA  Increase ST | Decrease MVPA  Increase LPA |
| 20 min | 0.55 | 0.66 | 0.87 | -0.79 | -0.90 | -1.08 |
| ES | 0.25 | 0.30 | 0.39 | -0.35 | -0.40 | -0.48 |
| 15 min | 0.43 | 0.51 | 0.67 | -0.56 | -0.64 | -0.78 |
| ES | 0.19 | 0.23 | 0.30 | -0.25 | -0.29 | -0.35 |
| 10 min | 0.30 | 0.35 | 0.45 | -0.36 | -0.41 | -0.50 |
| ES | 0.13 | 0.16 | 0.20 | -0.16 | -0.18 | -0.23 |
| 5 min | 0.16 | 0.18 | 0.23 | -0.17 | -0.20 | -0.24 |
| ES | 0.07 | 0.08 | 0.10 | -0.08 | -0.09 | -0.11 |
| Note. ES – Effect Size; ST – Sedentary Time; LPA – Light Physical Activity; MVPA – Moderate-to-Vigorous Physical Activity | | | | | | |

| **Secondary Schools (Boys)** | Estimated difference in Dragon Challenge Process Score | | | | | |
| --- | --- | --- | --- | --- | --- | --- |
| Time reallocations | Increase MVPA  Decrease Sleep | Increase MVPA  Decrease ST | Increase MVPA  Decrease LPA | Decrease MVPA  Increase Sleep | Decrease MVPA  Increase ST | Decrease MVPA  Increase LPA |
| 20 min | 0.72 | 0.68 | 0.64 | -1.08 | -1.03 | -1.00 |
| ES | 0.28 | 0.26 | 0.25 | -0.42 | -0.40 | -0.38 |
| 15 min | 0.56 | 0.53 | 0.50 | -0.75 | -0.72 | -0.69 |
| ES | 0.22 | 0.20 | 0.19 | -0.29 | -0.28 | -0.27 |
| 10 min | 0.39 | 0.37 | 0.35 | -0.47 | -0.45 | -0.43 |
| ES | 0.15 | 0.14 | 0.13 | -0.18 | -0.17 | -0.17 |
| 5 min | 0.20 | 0.19 | 0.18 | -0.22 | -0.21 | -0.20 |
| ES | 0.08 | 0.07 | 0.07 | -0.09 | -0.08 | -0.08 |
| Note. ES – Effect Size; ST – Sedentary Time; LPA – Light Physical Activity; MVPA – Moderate-to-Vigorous Physical Activity | | | | | | |
