# Supplementary material for: Cross-sectional associations between 24-hour activity behaviours and motor competence in youth: a compositional data analysis
Source: J Act Sedentary Sleep Behav. 2022 Sep 1;1:3. doi: 10.1186/s44167-022-00003-3 (PMC11934481; doi:10.1186/s44167-022-00003-3)
Supplement: Supplementary file 3 — Additional file 3. One for one time reallocations: which presents figures showing estimated difference in Dragon Challenge scores associated with time reallocation between pairs of behaviours. [file 44167_2022_3_MOESM3_ESM.docx]

Additional Material S3. One for one time reallocation figures for Dragon Challenge scores that were significantly associated with the activity compositions and the activity behaviour isometric log-ratio pivot coordinates.


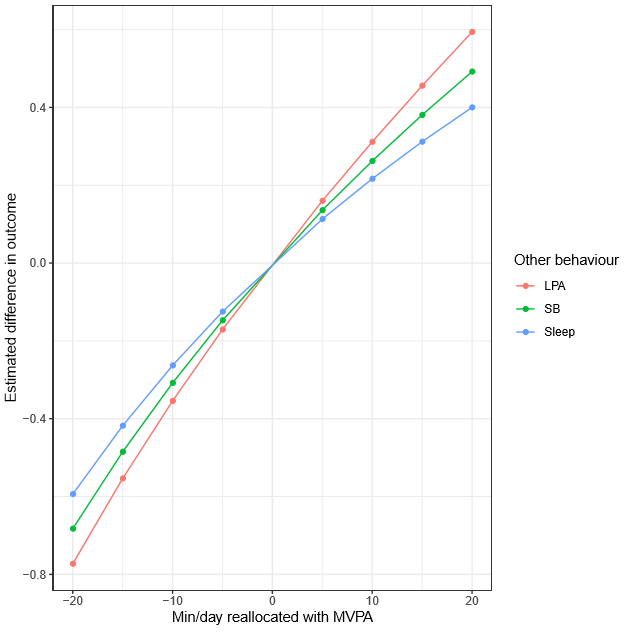


Figure S2. Estimated difference in Dragon Challenge scores associated with hypothetical time reallocation between pairs of behaviours: difference in all primary school participants’ time score (y-axis) associated with the difference in MVPA to each of the remaining behaviours. MVPA – Moderate-to-Vigorous Physical Activity; LPA – Light Physical Activity; SB – Sedentary Time/Behaviour.


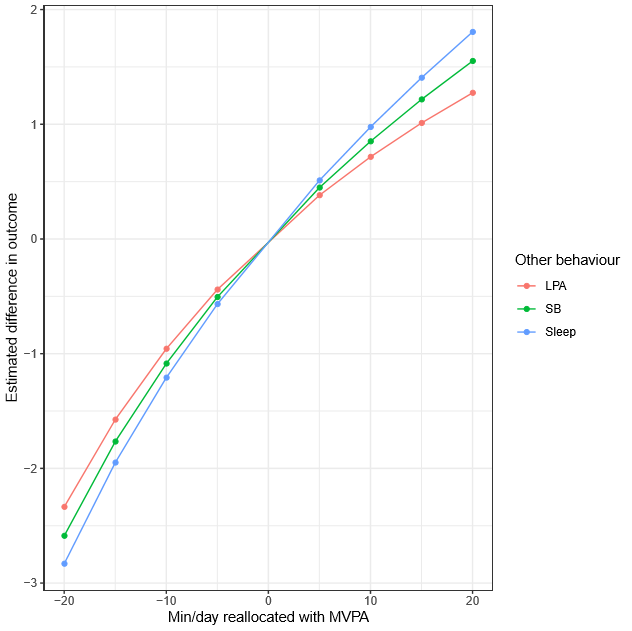

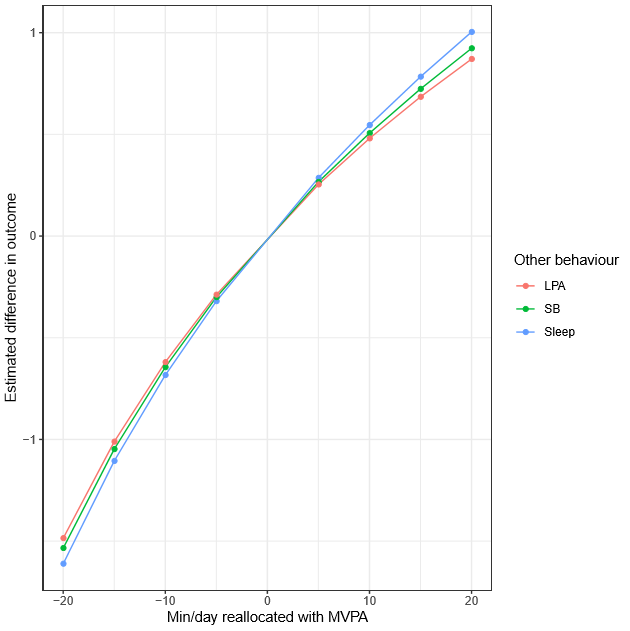

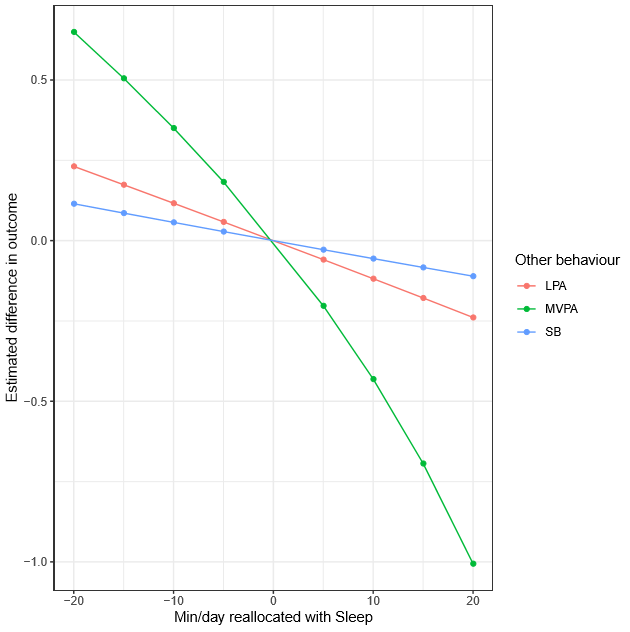


(c)

(b)

(a)

Figure S3a-c. Estimated difference in Dragon Challenge scores associated with hypothetical time reallocation between pairs of behaviours: difference in secondary school participants’ (a) overall and (b) process scores associated with the difference in MVPA to each of the remaining activities, and difference in (c) product score associated with the difference in sleep to each of the remaining activities. For example, adding 20 minutes to sleep at the expense of MVPA (green line) while keeping LPA and ST constant was associated with an estimated decrease of 1.01 in the product score (Figure S3c). y-axis for (S3a) shows unit increases/decreases in Dragon Challenge overall score, y-axis for (S3b) shows unit increases/decreases in Dragon Challenge process score, and y-axis for (S3c) shows unit increases/decreases in Dragon Challenge product score. MVPA – Moderate-to-Vigorous Physical Activity; LPA – Light Physical Activity; SB – Sedentary Time/Behaviour.


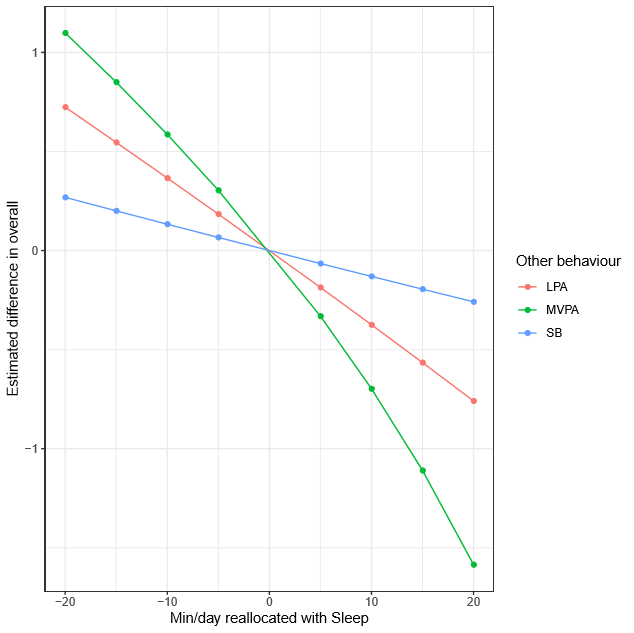

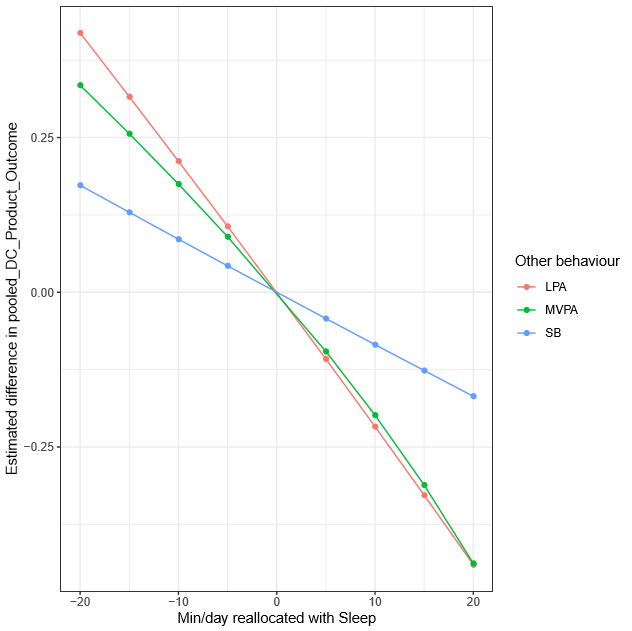


(b)

(a)


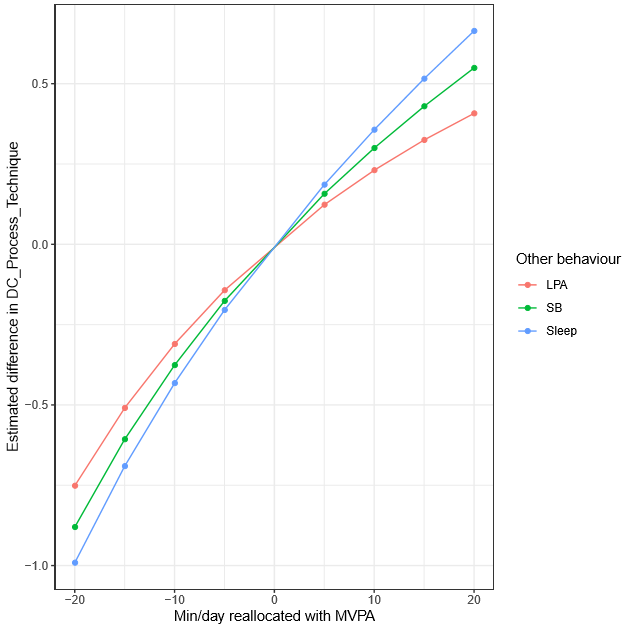


(c)

Figure S4a-c. Estimated difference in Dragon Challenge scores associated with hypothetical time reallocation between pairs of behaviours: difference in all girls’ (a) overall and (b) product scores associated with the difference in sleep to each of the remaining activities, and (c) process score associated with the difference in MVPA to each of the remaining activities. y-axis for (S4a) shows unit increases/decreases in Dragon Challenge overall score, y-axis for (S4b) shows unit increases/decreases in Dragon Challenge product score, and y-axis for (S4c) shows unit increases/decreases in Dragon Challenge process score. LPA – Light Physical Activity; MVPA – Moderate-to-Vigorous Physical Activity; SB – Sedentary Time/Behaviour.


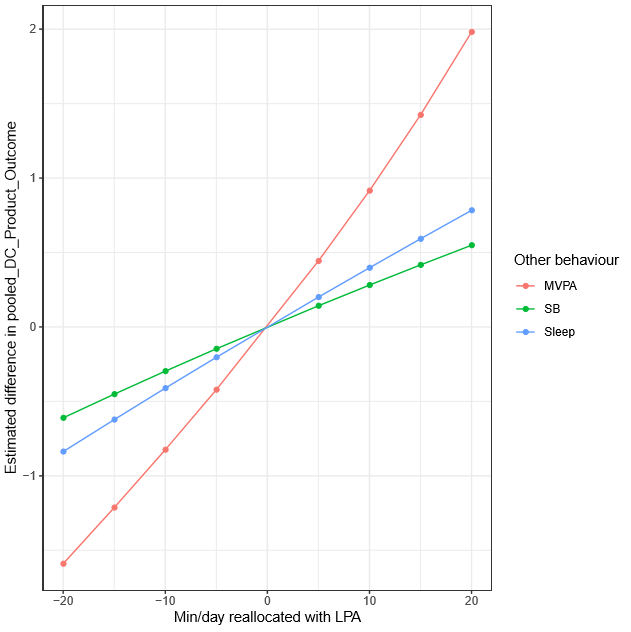


Figure S5. Estimated difference in Dragon Challenge scores associated with hypothetical time reallocation between pairs of behaviours: difference in primary school girls’ product score (y-axis) associated with the difference in LPA to each of the remaining behaviours. LPA – Light Physical Activity; MVPA – Moderate-to-Vigorous Physical Activity; SB – Sedentary Time/Behaviour.


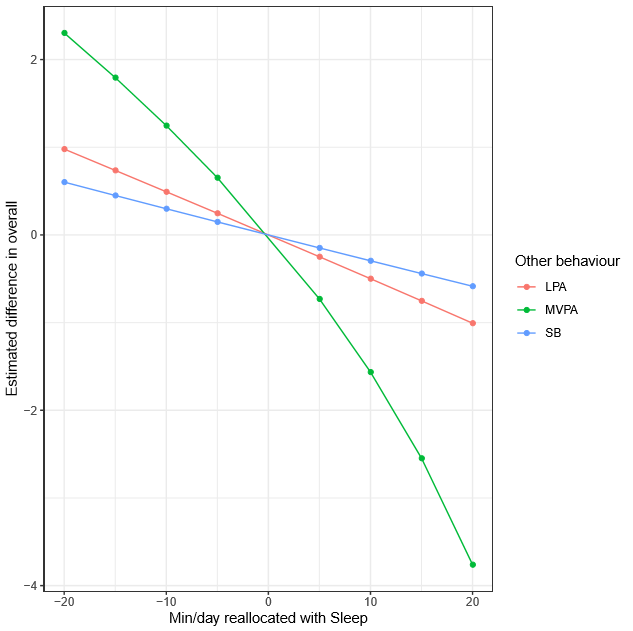

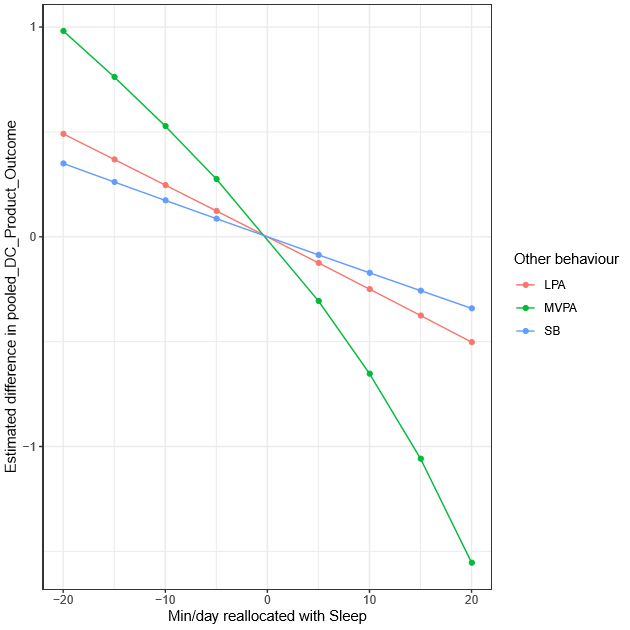


(b)

(a)


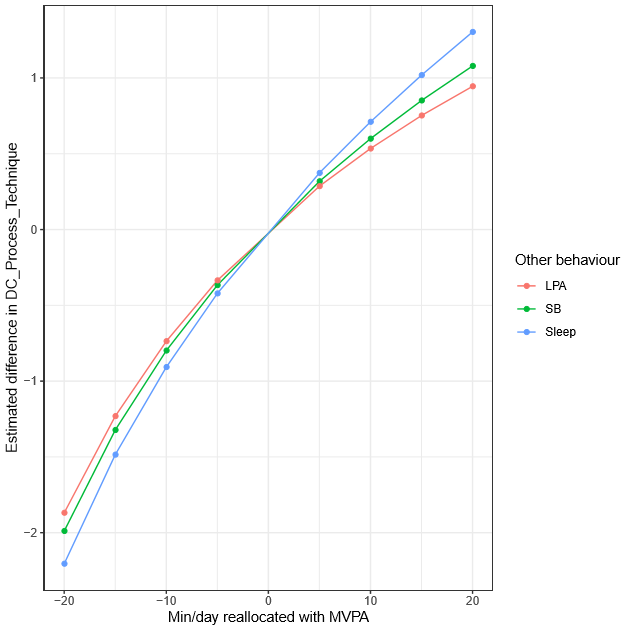


(c)

Figure S6a-c. Estimated difference in Dragon Challenge scores associated with hypothetical time reallocation between pairs of behaviours: difference in secondary school girls’ (a) overall and (b) product scores associated with the difference in sleep to each of the remaining activities, and (c) process score associated with the difference in MVPA to each of the remaining activities. y-axis for (S6a) shows unit increases/decreases in Dragon Challenge overall score, y-axis for (S6b) shows unit increases/decreases in Dragon Challenge product score, and y-axis for (S6c) shows unit increases/decreases in Dragon Challenge process score. LPA – Light Physical Activity; MVPA – Moderate-to-Vigorous Physical Activity; SB – Sedentary Time/Behaviour.


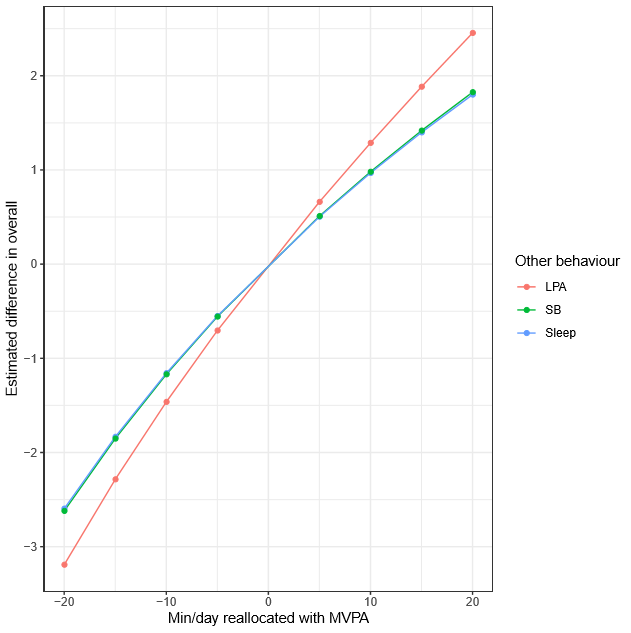

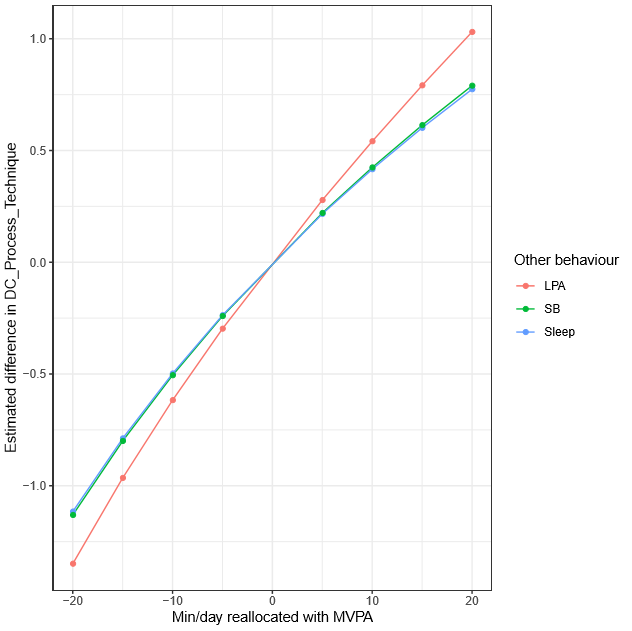


(b)

(a)


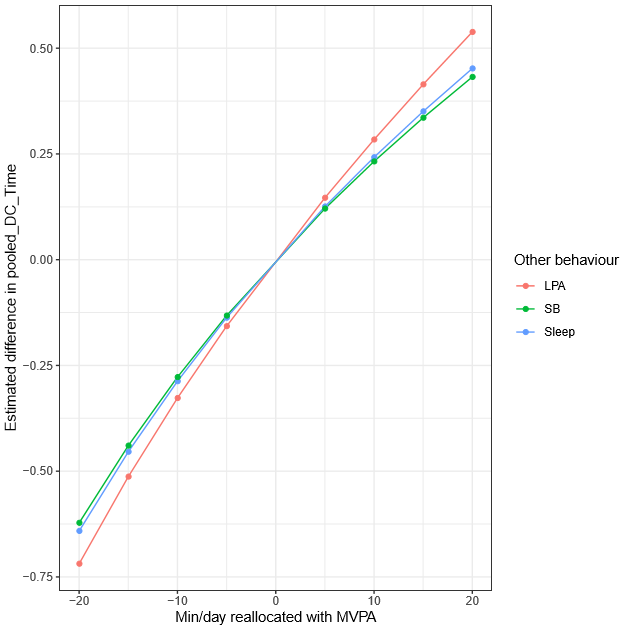


(c)

Figure S7a-c. Estimated difference in Dragon Challenge scores associated with hypothetical time reallocation between pairs of behaviours: difference in all boys’ (a) overall, (b) process, and (c) time scores associated with the difference in MVPA to each of the remaining activities. y-axis for (S7a) shows unit increases/decreases in Dragon Challenge overall score, y-axis for (S7b) shows unit increases/decreases in Dragon Challenge process score, and y-axis for (S7c) shows unit increases/decreases in Dragon Challenge time score. MVPA – Moderate-to-Vigorous Physical Activity; LPA – Light Physical Activity; SB – Sedentary Time/Behaviour.


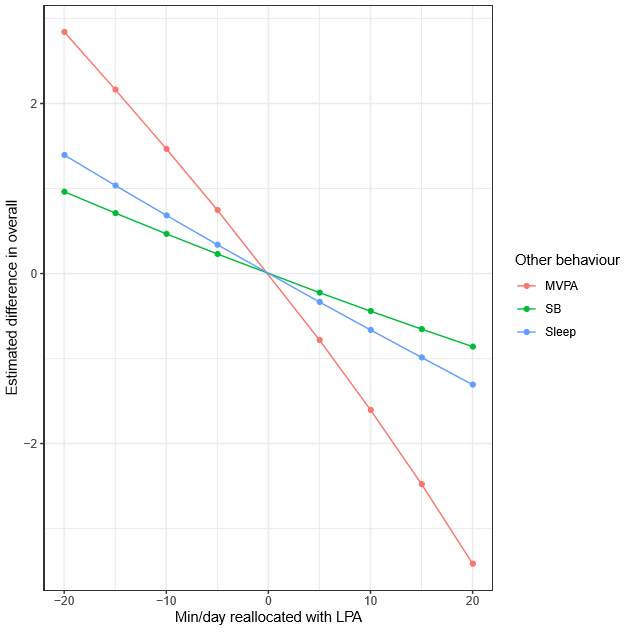

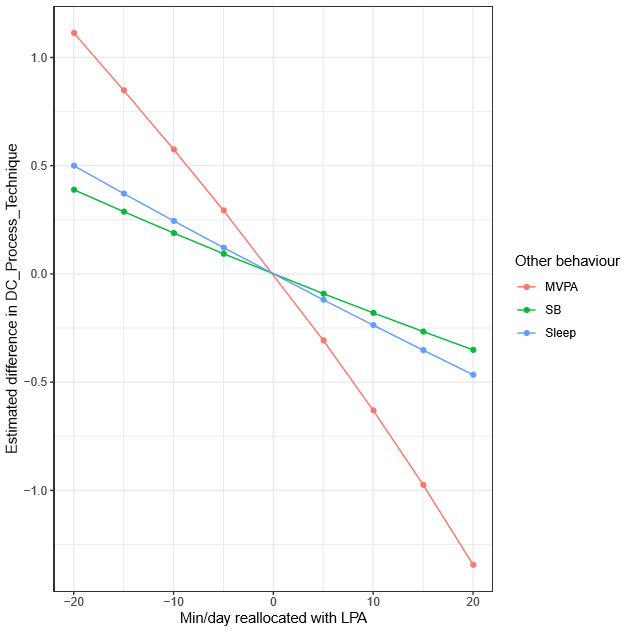


(b)

(a)


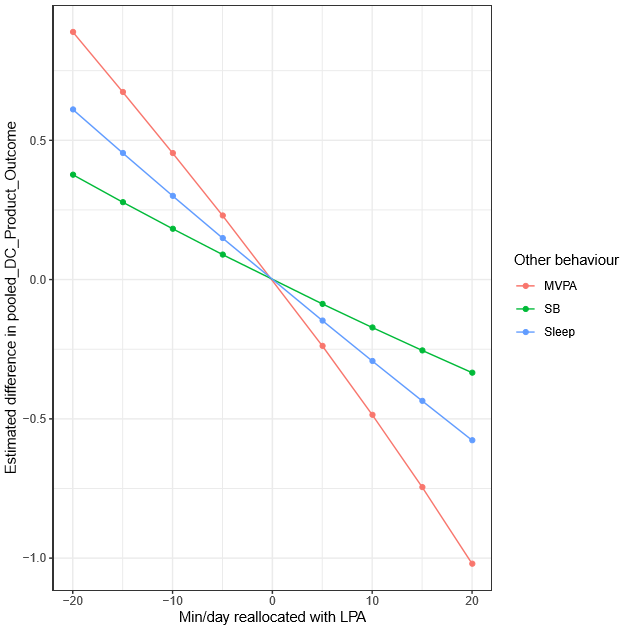

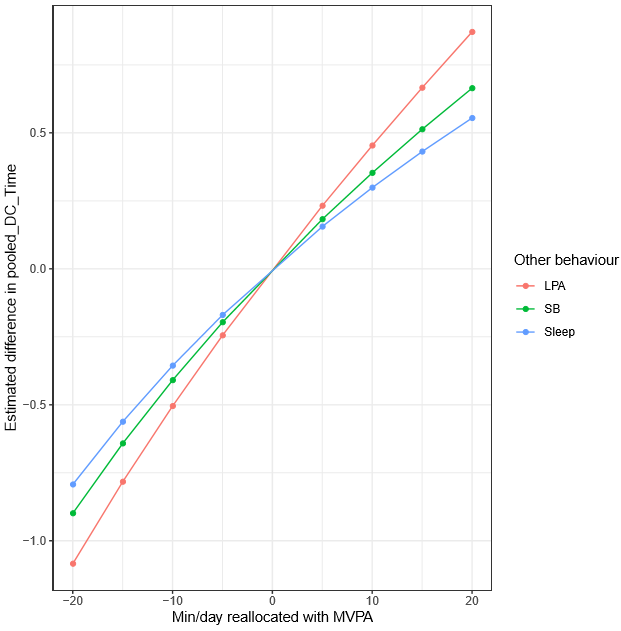


(d)

(c)

Figure S8a-d. Estimated difference in Dragon Challenge scores associated with hypothetical time reallocation between pairs of behaviours: difference in primary school boys’ (a) overall, (b) process, and (c) product scores associated with the difference in LPA to each of the remaining activities, and (d) time score associated with the difference in MVPA to each of the remaining activities. y-axis for (S8a) shows unit increases/decreases in Dragon Challenge overall score, y-axis for (S8b) shows unit increases/decreases in Dragon Challenge process score, y-axis for (S8c) shows unit increases/decreases in Dragon Challenge product score, and y-axis for (S8d) shows unit increases/decreases in Dragon Challenge time score. LPA – Light Physical Activity; MVPA – Moderate-to-Vigorous Physical Activity; SB – Sedentary Time/Behaviour.


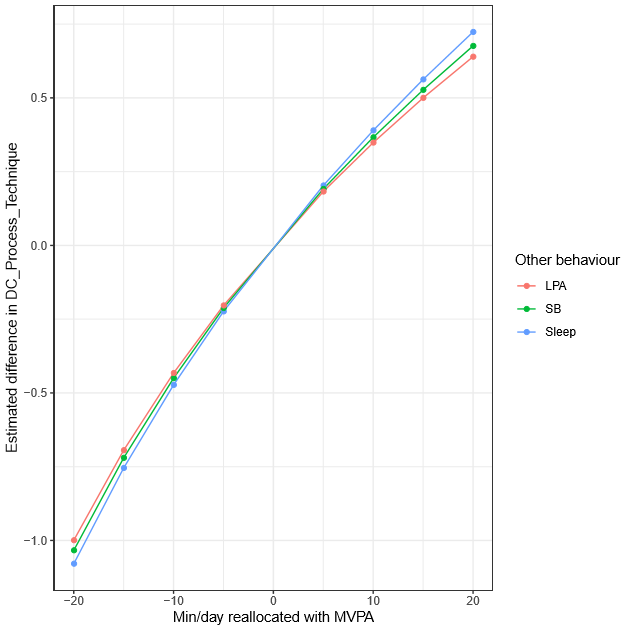


Figure S9. Estimated difference in Dragon Challenge scores associated with hypothetical time reallocation between pairs of behaviours: difference in secondary school boys’ process score (y-axis) associated with the difference in MVPA to each of the remaining behaviours. MVPA – Moderate-to-Vigorous Physical Activity; LPA – Light Physical Activity; SB – Sedentary Time/Behaviour.
