# Supplementary material for: Cross-sectional associations between 24-hour activity behaviours and motor competence in youth: a compositional data analysis
Source: J Act Sedentary Sleep Behav. 2022 Sep 1;1:3. doi: 10.1186/s44167-022-00003-3 (PMC11934481; doi:10.1186/s44167-022-00003-3)
Supplement: Supplementary file 4 — Additional file 4. Variation matrices: presents compositional variation matrices of time spent in sleep, ST, LPA, and MVPA. [file 44167_2022_3_MOESM4_ESM.docx]

Additional Material S4. Variation matrices of time spent in sleep, ST, LPA, and MVPA

|  | Sleep | ST | LPA | MVPA |
| --- | --- | --- | --- | --- |
| All participants |  |  |  |  |
| Sleep | 0.000 | 0.045 | 0.045 | 0.112 |
| ST | 0.045 | 0.000 | 0.066 | 0.223 |
| LPA | 0.045 | 0.066 | 0.000 | 0.096 |
| MVPA | 0.112 | 0.223 | 0.096 | 0.000 |
| Primary school participants |  |  |  |  |
| Sleep | 0.000 | 0.017 | 0.038 | 0.064 |
| ST | 0.017 | 0.000 | 0.061 | 0.122 |
| LPA | 0.038 | 0.061 | 0.000 | 0.049 |
| MVPA | 0.064 | 0.122 | 0.049 | 0.000 |
| Secondary school participants |  |  |  |  |
| Sleep | 0.000 | 0.026 | 0.044 | 0.202 |
| ST | 0.026 | 0.000 | 0.054 | 0.288 |
| LPA | 0.044 | 0.054 | 0.000 | 0.156 |
| MVPA | 0.202 | 0.288 | 0.156 | 0.000 |

Note. ST – Sedentary Time; LPA – Light Physical Activity; MVPA – Moderate-to-Vigorous

Physical Activity
